# Comparison of UK paediatric SARS-CoV-2 admissions across the first and second pandemic waves

**DOI:** 10.1038/s41390-022-02052-5

**Published:** 2022-04-22

**Authors:** Olivia V. Swann, Louisa Pollock, Karl A. Holden, Alasdair P. S. Munro, Aisleen Bennett, Thomas C. Williams, Lance Turtle, Cameron J. Fairfield, Thomas M. Drake, Saul N. Faust, Ian P. Sinha, Damian Roland, Elizabeth Whittaker, Shamez N. Ladhani, Jonathan S. Nguyen-Van-Tam, Michelle Girvan, Chloe Donohue, Cara Donegan, Rebecca G. Spencer, Hayley E. Hardwick, Peter J. M. Openshaw, J. Kenneth Baillie, Ewen M. Harrison, Annemarie B. Docherty, Malcolm G. Semple, on behalf of ISARIC4C Investigators

**Affiliations:** 1 Department of Child Life and Health, University of Edinburgh, Edinburgh, UK; 2 Paediatric Infectious Diseases, Royal Hospital for Children, Glasgow, UK; 3 Child Health, School of Medicine, Dentistry & Nursing, University of Glasgow, Glasgow, UK; 4 NIHR Health Protection Research Unit in Emerging and Zoonotic Infections, Institute of Infection, Veterinary and Ecological Sciences, Faculty of Health and Life Sciences, University of Liverpool, Liverpool, L69 7BE UK; 5 Respiratory Medicine, Alder Hey Children’s NHS Foundation Trust, Liverpool, UK; 6 NIHR Southampton Clinical Research Facility and NIHR Biomedical Research Centre, University Hospital Southampton NHS Foundation Trust, Southampton, UK; 7 Faculty of Medicine and Institute for Life Sciences, University of Southampton, Southampton, UK; 8 Institute of Infection and Immunity, St George’s, University of London, London, UK; 9 Tropical and Infectious Diseases Unit, Liverpool University Hospitals NHS Foundation Trust, Member of Liverpool Health Partners, Liverpool, UK; 10 Centre for Medical Informatics, Usher Institute, University of Edinburgh, Edinburgh, UK; 11 Women’s and Children’s Health, Institute of Life Course and Medical Sciences, Faculty of Health and Life Sciences, University of Liverpool, Liverpool, UK; 12 Paediatric Emergency Medicine Leicester Academic (PEMLA) Group, University Hospitals of Leicester NHS Trust, Leicester, UK; 13 SAPPHIRE Group, Health Sciences, Leicester University, Leicester, UK; 14 Section of Paediatric Infectious Diseases, Imperial College London, London, UK; 15 Paediatric Infectious Diseases, Imperial College Healthcare National Health System Trust, London, UK; 16 Immunisation and Countermeasures Division, Public Health England Colindale, London, UK; 17 Paediatric Infectious Disease, St. George’s Hospital London, London, UK; 18 Division of Epidemiology and Public Health, University of Nottingham School of Medicine, Nottingham, UK; 19 Liverpool Clinical Trials Centre, University of Liverpool, Liverpool, UK; 20 National Heart and Lung Institute, Imperial College London, London, UK; 21 Roslin Institute, University of Edinburgh, Edinburgh, UK; 22 Intensive Care Unit, Royal Infirmary Edinburgh, Edinburgh, UK

## Abstract

**Background:**

We hypothesised that the clinical characteristics of hospitalised children and young people (CYP) with SARS-CoV-2 in the UK second wave (W2) would differ from the first wave (W1) due to the alpha variant (B.1.1.7), school reopening and relaxation of shielding.

**Methods:**

Prospective multicentre observational cohort study of patients <19 years hospitalised in the UK with SARS-CoV-2 between 17/01/20 and 31/01/21. Clinical characteristics were compared between W1 and W2 (W1 = 17/01/20-31/07/20,W2 = 01/08/20-31/01/21).

**Results:**

2044 CYP < 19 years from 187 hospitals. 427/2044 (20.6%) with asymptomatic/incidental SARS-CoV-2 were excluded from main analysis. 16.0% (248/1548) of symptomatic CYP were admitted to critical care and 0.8% (12/1504) died. 5.6% (91/1617) of symptomatic CYP had Multisystem Inflammatory Syndrome in Children (MIS-C). After excluding CYP with MIS-C, patients in W2 had lower Paediatric Early Warning Scores (PEWS, composite vital sign score), lower antibiotic use and less respiratory and cardiovascular support than W1. The proportion of CYP admitted to critical care was unchanged. 58.0% (938/1617) of symptomatic CYP had no reported comorbidity. Patients without co-morbidities were younger (42.4%, 398/938, <1 year), had lower PEWS, shorter length of stay and less respiratory support.

**Conclusions:**

We found no evidence of increased disease severity in W2 vs W1. A large proportion of hospitalised CYP had no comorbidity.

**Impact:**

- No evidence of increased severity of COVID-19 admissions amongst children and young people (CYP) in the second vs first wave in the UK, despite changes in variant, relaxation of shielding and return to face-to-face schooling.
- CYP with no comorbidities made up a significant proportion of those admitted. However, they had shorter length of stays and lower treatment requirements than CYP with comorbidities once those with MIS-C were excluded.
- At least 20% of CYP admitted in this cohort had asymptomatic/incidental SARS-CoV-2 infection.
- This paper was presented to SAGE to inform CYP vaccination policy in the UK.

## Introduction

Children and young people (CYP) were significantly less affected than adults during the first wave of the COVID-19 pandemic, with regards to case numbers, disease severity, hospital admissions and death.^1–4^ The reasons for the predominantly mild disease course in CYP are not yet well defined, although several hypotheses have been proposed, including reduced expression of ACE2 (the binding receptor for SARS-CoV-2) in the lower airways, immunity from prior exposure to seasonal coronaviruses and difference in immune response to acute SARS-CoV-2 infection.^5^ Whilst the clinical profile of CYP with symptomatic SARS-CoV-2 infection (COVID-19) shares similarities with other respiratory viruses^2,3^ (with at-risk cohorts including young infants and those with neurological and cardiac comorbidities^2,4^), the virus also has unusual presentations in the paediatric population. A small proportion of CYP exposed to the virus go on to develop a severe hyperinflammatory syndrome^2^ known as multisystem inflammatory syndrome in children (MIS-C), also known as paediatric inflammatory multisystem syndrome temporally associated with SARS-CoV-2 (PIMS-TS), which shares features of Kawasaki disease and Toxic Shock Syndrome, and often requires management in intensive care.^6,7^

In the United Kingdom, the SARS-CoV-2 pandemic has been considered as a series of waves with the first wave spanning the beginning of March 2020 to the end of May 2020 (peaking in early April) and the second wave from the beginning of September 2020 to the end of April 2021 (peaking in early January).^8^ A significant amount of knowledge was gained about the clinical characteristics and outcomes of COVID-19 in CYP during the first wave of the pandemic, however, several external factors changed with the emergence of the second wave. Most UK schools were closed during the first pandemic wave in the spring and summer of 2020, with a few remaining open for children of key workers but were mostly open during the subsequent autumn and winter wave of infection (Supp Fig. A). This policy reflected the view that the educational, social, health and economic benefits of in-person schooling outweighed the harms associated with school transmission of SARS-CoV-2 at that time. During the first wave, some CYP were identified as extremely clinically vulnerable and advised to shield from all non-essential contact. This advice was removed in autumn 2020. New variants have emerged, including the alpha variant (B.1.1.7) first detected in Southeast England in September 2020, becoming the predominant variant throughout the UK by the end of December.^9,10^ The alpha variant contains mutations that permit some immune escape^11^ in those who had been previously infected, with increased transmissibility^12^ and more severe disease with higher rates of hospitalisation and death in adults.^13^

The emergence of the alpha variant in England also led to concerns of increased transmissibility in CYP as they formed a higher proportion of total cases in England when compared to the first pandemic wave.^14^ This may have been due to the emergence of the variant coinciding with a period when schools were open and subject to increased testing, but the rest of UK society was in “lockdown”.^12^ Whether the alpha variant, dominant in the second wave, causes different symptoms or more severe disease in CYP compared to strains circulating in the first wave has not been analysed in detail.

We test the hypothesis that clinical characteristics of hospitalised children with SARS-CoV-2 in the UK second wave would differ from the first due to the combined impact of the alpha variant, school reopening and relaxation of shielding.

We aimed to characterise and compare the clinical features and outcomes of CYP aged <19 years who were hospitalised with SARS-CoV-2 infection during the first and second waves across England, Scotland, and Wales from 17th January 2020 to 31st January 2021, as part of the International Severe Acute Respiratory and Emerging Infection Consortium -World Health Organisation, Clinical Characterisation Protocol in the United Kingdom (ISARIC WHO CCP-UK).

## Methods

### Study design, setting and participants

The protocol, associated documents, and details of the Independent Data and Material Access Committee (IDAMAC) are available at https://isaric4c.net.^15^ We included all patients aged <19 years with clinician-reported SARS-CoV-2 infection who were enroled into the ongoing, prospective ISARIC WHO CCP-UK cohort study involving National Health Service (NHS) hospitals in England, Wales, and Scotland between 17th January 2020 to 31st January 2021 who had at least 2 weeks of outcome data available (Wave 1—17th January 2020–31st July 2020, Wave 2—1st August 2020–31st January 2021).^16^ Patients were managed by their local clinicians and participation in this study had no influence on management. We used the Strengthening the Reporting of Observational Studies in Epidemiology guidelines for reporting this observational study.

### Data collection

Data were collected from healthcare records onto case report forms through a secure online database, REDCap (Research Electronic Data Capture, Vanderbilt University, hosted by the University of Oxford, UK). Demographic (including age, sex, self-reported ethnicity, postal code) and baseline data (including comorbidities and regular medications taken) alongside data on symptoms, clinical signs, laboratory and pathology investigations, and treatments received while admitted were collected. Centres also recorded whether their team had treated patients as having MIS-C.

### Clinician-reported SARS-CoV-2

Patients were included in this report if the study team had reported them as ‘laboratory-proven’ SARS-CoV-2. Where patients were reported as ‘suspected’ SARS-CoV-2, the patients’ virological data were reviewed, and patients were included if there was documented evidence of a positive PCR, serology or lateral-flow antigen testing for SARS-CoV-2 (usually self-administered in the community).

### ‘Incidental SARS-CoV-2’ and ‘other reason for admission’ variables

We reviewed all available free text for evidence of incidental SARS-CoV-2 infection (e.g., hospitalisation for elective surgery, road traffic accidents, or drug overdoses, see *Supplementary Methods*). Patients in whom SARS-CoV-2 was judged to be incidental or who were asymptomatic at the time of assessment for SARS-CoV-2 were censored from the main analysis.

### Symptomatic patients with SARS-CoV-2

Patients who had reported symptoms together with those missing symptom data are referred to as ‘symptomatic CYP,’ however, it is possible that not all symptoms were due to SARS-CoV-2.

### Paediatric Early Warning Score (PEWS)

PEWS (a composite score of vital signs for early recognition of unwell patients) was used as a measure of disease severity at admission.^17^

### Outcomes

The primary outcomes of this study were admission to critical care (high dependency units (HDUs) or paediatric intensive care units (PICUs)), development of MIS-C, and in-hospital mortality for CYP and young people with symptomatic SARS-CoV-2 infection (COVID-19). We also examined the need for any respiratory and cardiovascular (inotropic) support.

### Bias

As specialist children’s hospitals (tertiary centres) are more likely to have both paediatric critical care facilities and paediatric research teams with capacity to support participating in the study, it is possible that CYP admitted to these centres are over-represented. We compared the proportion of CYP who were reported from hospitals with onsite access to a PICU to ascertain whether this differed between the waves, potentially influencing the severity of patients reported (see *Supplementary Results*).

To explore how well the ISARIC data reflected regional variations in SARS-CoV-2 prevalence, we also compared the number of CYP reported to ISARIC against the numbers of local SARS-CoV-2 cases identified by pillar 1 (hospital) and pillar 2 (community) testing across NHS regions (see *Supplementary Results*).

Our previous analysis identified a peak of MIS-C cases occurring approximately four weeks after the peak of CYP admissions in the first wave, with patients with MIS-C being five times more likely to be admitted to critical care.^2^ The data collection for this current analysis ended on 31^st^ Jan 2021, after the peak of the second wave but before its end (estimated end of April 2021). As such, given the time lag in the presentation of MIS-C, we anticipated the number of cases of MIS-C in the second wave would be an underestimate. This could bias towards fewer severely ill CYP in the second wave. To reduce this bias, patients with MIS-C were censored for analyses comparing disease severity or treatments received across waves 1 and 2, but retained for whole cohort analyses.

### Statistical analysis

Continuous variables are displayed as means (standard deviations) or if non-normally distributed as medians (interquartile ranges). Categorical variables are presented as frequencies (percentages) unless otherwise stated. For univariable comparisons, we used Welch’s *t*, analysis of variance, Mann-Whitney *U*, or Kruskal-Wallis tests, according to data distribution. We compared categorical data by using χ^2^ tests and considered a *p* value below 0.05 to be statistically significant. All tests were two sided and we made no adjustment for multiple comparisons. A directed acyclic graph was constructed prior to undertaking a mixed effect multivariable analysis (Supp Fig. B). Hospital was included as a random effect in the multivariable analysis. Parsimonious criterion-based model building used the following principles: relevant explanatory variables were identified from previous studies; interactions were checked at first order level; final model selection was informed by the Akaike information criterion and C statistic, with appropriate assumptions checked including the distribution of residuals. We used R (R Core Team version 3.6.3, Vienna, Austria) for statistical analyses, with packages including tidyverse, finalfit lubridate, UpSetR and ggplot2.

### Patient and public involvement

Patients and the public were not involved in the design, conduct, or reporting of this rapid response research which is part of an ongoing urgent public health research study, however their involvement is in now progress.

### Legal basis for data collection and ethics approval

In England and Wales routine anonymised data from medical records was collected without the need for consent under regulation 3 (4) of the Health Service (Control of Patient Information) Regulations 2002. In Scotland, a waiver of need for consent was obtained from the Public Benefit and Privacy Panel. Ethical approval was given by the South Central–Oxford C Research Ethics Committee in England (reference 13/SC/0149) and the Scotland A Research Ethics Committee (reference 20/SS/0028).

## Results

**Fig. 1:**
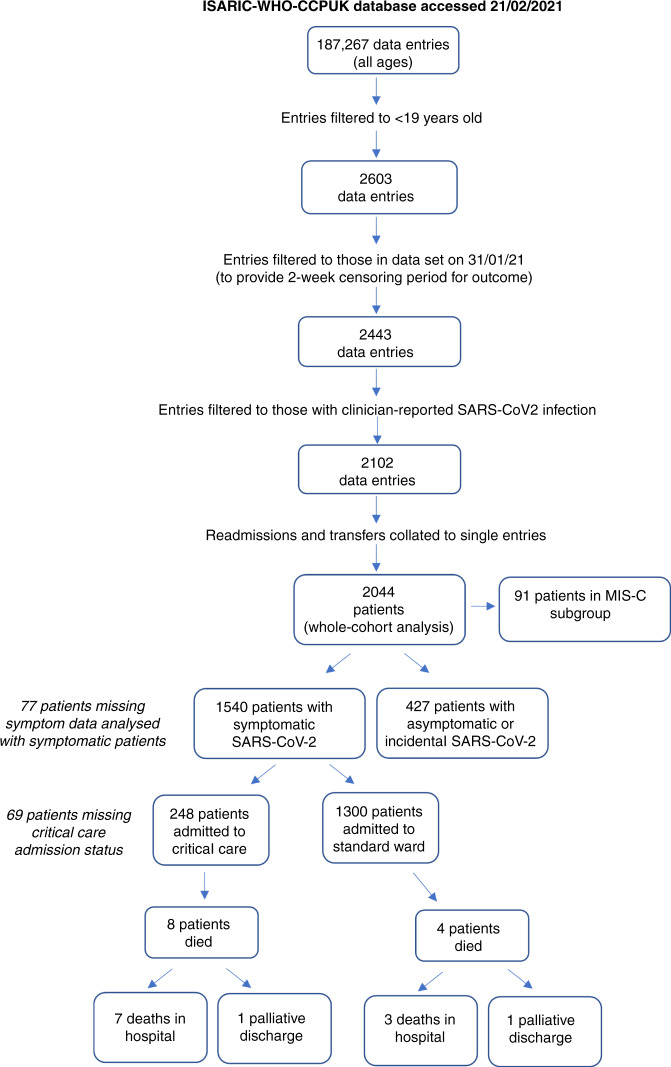
Flowchart of patient inclusion and outcomes.

Between 17th January 2020 and 31st January 2021, 187,267 admissions of all ages were enrolled. There were 2044 (1.1%) CYP aged <19 years of age with clinician-reported SARS-CoV-2 infection reported from 187 hospitals across England, Scotland, and Wales. Of these, 1540 (75.3%) had symptoms at presentation, 427 (20.6%) had asymptomatic or incidental SARS-CoV-2 infection and 77 (3.8%) were missing data on symptoms (Fig. 1). Of the symptomatic CYP, 91 were identified as having MIS-C.

### Demographics of symptomatic CYP admitted in the first wave vs second wave

In total, 764 CYP were admitted during wave one (W1, 17th January to 31st July 2020) and 1280 during wave two (W2, 1st August 2020 to 31st January 2021). CYP in W2 were significantly older (median age 6.5 years, IQR 0.3–14.9) than W1 (4.0 (0.4–13.6), *p* = 0.015) *(*Table 1*)*. CYP of South Asian ethnicity were over-represented in W2 (19.1%, 155/810) compared to W1 (13.6%, 78/575, *p* = 0.008). W2 saw a lower proportion of likely hospital-acquired SARS-CoV-2 (2.2% (21/952) vs W1, 6.9% (46/665), *p* < 0.001). Fever was more common in W1 (76.8% (491/639) vs 63.6% (544/855), *p* < 0.001, Supp Table B), otherwise presenting symptoms were very similar (Supp Fig. E). Comorbidities were similar in W2 and W1 for symptomatic CYP (Supp Table C) and the whole cohort (Supp Table D).

**Table 1:** Demographics of patients <19 years by wave of SARS-CoV-2 pandemic (excluding patients with asymptomatic and incidental SARS-CoV-2).

	Total *N*		First	Second	*p*
Total *N* (%)			665 (41.1)	952 (58.9)	
Age at assessment (Years)	1617 (100.0)	Median (IQR)	4.0 (0.4–13.6)	6.5 (0.3–14.9)	0.015
Age	1617 (100.0)	<1 mth	48 (7.2)	74 (7.8)	0.011
		>1mth < 1 y	181 (27.2)	226 (23.7)	
		1–4 y	117 (17.6)	139 (14.6)	
		5–9 y	90 (13.5)	101 (10.6)	
		10–14 y	102 (15.3)	182 (19.1)	
		15–19 y	127 (19.1)	230 (24.2)	
Sex at Birth	1613 (99.8)	Male	363 (54.6)	500 (52.5)	0.463
		Female	301 (45.3)	449 (47.2)	
		(Missing)	1 (0.2)	3 (0.3)	
Ethnicity	1385 (85.7)	White	330 (49.6)	472 (49.6)	0.008
		Black	49 (7.4)	56 (5.9)	
		South Asian	78 (11.7)	155 (16.3)	
		Other ethnic minority	118 (17.7)	127 (13.3)	
		(Missing)	90 (13.5)	142 (14.9)	
IMD quintile	1491 (92.2)	1 (most deprived)	212 (31.9)	330 (34.7)	0.224
		2	130 (19.5)	180 (18.9)	
		3	87 (13.1)	144 (15.1)	
		4	82 (12.3)	113 (11.9)	
		5 (least deprived)	101 (15.2)	112 (11.8)	
		(Missing)	53 (8.0)	73 (7.7)	
Potential hospital acquired SARS-CoV-2	1617 (100.0)	No	619 (93.1)	931 (97.8)	<0.001
		Yes	46 (6.9)	21 (2.2)	
Any comorbidity	1617 (100.0)	No/Unknown	367 (55.2)	571 (60.0)	0.062
		Yes	298 (44.8)	381 (40.0)	

First wave ending 31st July 2020. Values are numbers (percentages) unless stated otherwise.

*IQR* interquartile range, *IMD* Indices of multiple deprivation.

### Severity at presentation, treatments received and outcomes in symptomatic CYP (excluding MIS-C) examined by wave

Paediatric Early Warning Score (PEWS) at presentation was lower in W2 than W1, with 41.2% (343/832) of CYP in W2 having a PEWS > 2 at presentation vs 48.9% in W1 (291/595, *p* = 0.005, Table 2 and Supp. Fig F). Median length of stay was very short at 2 days (IQR 1–4) for both waves (Supp Fig. G). We found no difference in the proportion of symptomatic CYP admitted to critical care in W1 vs W2 (12.9% (78/604) vs (12.7% (109/855, *p* = 0.989, Table 2). CYP in W2 had lower antibiotic use than W1 (58.0% (467/806) vs 70.6% (415/588, *p* < 0.001)), were less likely to receive high flow oxygen, non-invasive or invasive respiratory support as well as fewer patients in W2 requiring inotropic support (1.0% (8/780) vs 3.6% (21/580, *p* = 0.002)). CYP in W2 were more likely to receive oral steroids. These associations persisted in a sensitivity analysis of the whole cohort (Supp Table E).

**Table 2:** Comparison of treatments received and outcomes by wave (excluding asymptomatic and incidental SARS-CoV-2 infections and patients with Multisystem Inflammatory Syndrome in Children (MIS-C)).

	Total *N*		First	Second	*p*
Total N (%)			616 (40.4)	910 (59.6)	
Antibiotic medication	1394 (91.3)	No	173 (28.1)	339 (37.3)	<0.001
		Yes	415 (67.4)	467 (51.3)	
		(Missing)	28 (4.5)	104 (11.4)	
Antiviral	1385 (90.8)	No	539 (87.5)	759 (83.4)	0.183
		Yes	43 (7.0)	44 (4.8)	
		(Missing)	34 (5.5)	107 (11.8)	
Maximal steroid therapy	1350 (88.5)	None	505 (82.0)	656 (72.1)	<0.001
		Oral	31 (5.0)	120 (13.2)	
		IV	15 (2.4)	23 (2.5)	
		(Missing)	65 (10.6)	111 (12.2)	
Maximum respiratory support	1459 (95.6)	No respiratory support	450 (73.1)	669 (73.5)	0.024
		Supplemental oxygen	60 (9.7)	99 (10.9)	
		High flow support	31 (5.0)	31 (3.4)	
		Non-invasive	25 (4.1)	24 (2.6)	
		Invasive	39 (6.3)	31 (3.4)	
		(Missing)	11 (1.8)	56 (6.2)	
ICU/HDU admission	1459 (95.6)	No	526 (85.4)	746 (82.0)	0.989
		Yes	78 (12.7)	109 (12.0)	
		(Missing)	12 (1.9)	55 (6.0)	
Inotrope	1360 (89.1)	No	559 (90.7)	772 (84.8)	0.002
		Yes	21 (3.4)	8 (0.9)	
		(Missing)	36 (5.8)	130 (14.3)	
PEWS over 2	1427 (93.5)	No	304 (49.4)	489 (53.7)	0.005
		Yes	291 (47.2)	343 (37.7)	
		(Missing)	21 (3.4)	78 (8.6)	
Length of stay	1301 (85.3)	Median (IQR)	2.0 (1.0–4.0)	2.0 (1.0–4.0)	0.079

*IQR* interquartile range, *ICU* intensive care unit, *HDU* high dependency unit, *PEWS* Paediatric Early Warning Score.

### CYP with MIS-C

There were 163 potential MIS-C patients, of whom 91 were confirmed by sites, 46 had other diagnoses and there was no response for 26 patients (Supp Fig. H). There was no significant difference in PEWS at presentation for patients with MIS-C between W1 and W2, but length of stay was shorter in W2 compared to W1 (median 6.0 days (4.0–10.0) vs 8.5 (5.8–12.0, *p* = 0.031). CYP with MIS-C in W2 were less likely to receive IVIg than in W1 (59.5% (25/42) vs 83.7% (41/49), *p* = 0.018, Supp Table F), but there was no difference in use of antibiotics, steroids (oral or IV), immunomodulators, respiratory or cardiac support or critical care admission.

### Factors associated with critical care admission

We reviewed the demographics and key clinical characteristics of CYP admitted to critical care, excluding those with asymptomatic or incidental SARS-CoV-2 infection (but including those with MIS-C). On univariable analysis, age groups 5-9 and 10-14 years were associated with critical care admission, as was non-white ethnicity, hospital-acquired SARS-CoV-2 infection, PEWS > 2 at admission and presence of an underlying comorbidity (Supp Table G). Of the 248 children admitted to critical care, 58.9% (146/248) were aged ≤11 years, i.e., in an age group with no current licenced vaccine available. On detailed review, comorbidities associated with critical care admission included prematurity, neurological comorbidity, neurodisability, respiratory comorbidity (excluding asthma) and cardiac comorbidities (Supp Table H). Whilst the majority of CYP admitted to critical care with symptomatic SARS-CoV-2 had comorbidities, 45.2% (112/248) had no reported comorbidity.

### CYP in W2 were no more likely to be admitted to critical care than W1 after excluding MIS-C

**Fig. 2:**
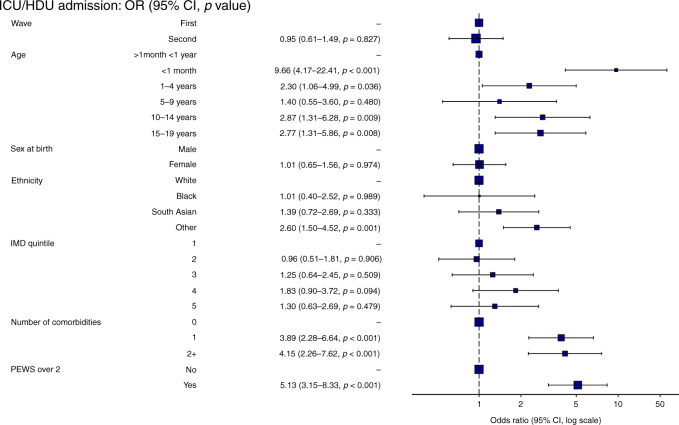
Forest plot of factors associated with admission to critical care unit (excluding asymptomatic and incidental SARSCoV-2 infections and patients with Multisystem Inflammatory Syndrome in Children (MIS-C)). Other = Other ethnic minorities, IMD Indices of multiple deprivation (1 = most deprived, 5 = least deprived), PEWS Paediatric early warning score at presentation, CI 95% confidence interval.

As our analysis period likely underestimates the proportion of CYP with MIS-C in W2 (see *Methods*), these patients were excluded from the multivariable analysis to reduce bias when comparing severity between the waves. Neonates and CYP aged 10-14 years and 15-19 years were more likely to be admitted to critical care (Fig. 2 and Supp Table I). Other ethnic minorities (i.e., not white, black or South-Asian) were significantly associated with critical care admission as was the presence of one or more comorbidities, and a PEWS of ≥2 at presentation. No association was seen between indices of multiple deprivation (IMD) or sex and admission to critical care. After taking patient demographics, comorbidity count, and PEWS score at presentation into account, we found that CYP were no more likely to be admitted to critical care in W2 when compared to W1.

### Factors associated with death

Outcome data were available for 1504/1617 symptomatic CYP where we identified 12 deaths (10 in hospital and 2 palliative discharges) and an overall mortality rate of 0.8% (12/1504). Information was available for 11 of these deaths. All 11 had significant comorbidities (severe neurodisability, malignancy, very premature, complex congenital heart disease, bacterial sepsis and complex life-limiting comorbidities). Five were <5 years old and six were >15 years old.

### Symptomatic patients with and without reported comorbidities (including those with MIS-C)

Patients without a reported comorbidity made up 58.0% (938/1617) of the symptomatic cohort (Supp Table J). Of CYP without a reported comorbidity, 70.4% (660/938) were ≤11 years (i.e. in an age group with no current licenced vaccine available) and 42.4% (398/938) were aged under 1 year. Among CYP ≥ 12 years, 47.5% (278/585) had no comorbidity recorded on admission. Patients without reported comorbidities had a lower PEWS score at presentation than those with comorbidities (2.0 (1.0–4.0) vs 2.0 (1.0–5.0), *p* = 0.014), and a shorter length of stay (2.0 days (1.0–3.0) vs 3.0 days (1.0–7.0), *p* < 0.001). CYP with no reported comorbidities were less likely to receive antiviral therapy, steroids, and all forms of respiratory support than in CYP with comorbidities (Table 3). Whilst the majority of CYP with no reported comorbidities received ward-level care, 12.9% (112/876) were admitted to critical care, however only 6.6% (58/876) required invasive or non-invasive ventilation. A sensitivity analysis showed that the rates of critical care admission, invasive and non-invasive ventilation, IV steroids and inotropes in CYP without reported comorbidities were driven by the subgroup with MIS-C (Supp Table K).

**Table 3:** Treatments received stratified by comorbidity (patients with asymptomatic or incidental SARS-CoV-2 infections excluded).

	Total *N*		No/Unknown comorbidity	Comorbidity present	*p*
Total *N* (%)			938 (58.0)	679 (42.0)	
Antibiotic medication	1477 (91.3)	No	301 (32.1)	216 (31.8)	0.273
		Yes	529 (56.4)	431 (63.5)	
		(Missing)	108 (11.5)	32 (4.7)	
Antiviral	1476 (91.3)	No	787 (83.9)	595 (87.6)	0.027
		Yes	42 (4.5)	52 (7.7)	
		(Missing)	109 (11.6)	32 (4.7)	
Maximal steroid therapy	1422 (87.9)	None	713 (76.0)	475 (70.0)	<0.001
		Oral	53 (5.7)	103 (15.2)	
		IV	42 (4.5)	36 (5.3)	
		(Missing)	130 (13.9)	65 (9.6)	
Maximum respiratory support	1550 (95.9)	No respiratory support	738 (78.7)	427 (62.9)	<0.001
		Supplemental oxygen	59 (6.3)	108 (15.9)	
		High flow support	21 (2.2)	47 (6.9)	
		Non-invasive	25 (2.7)	35 (5.2)	
		Invasive	33 (3.5)	57 (8.4)	
		(Missing)	62 (6.6)	5 (0.7)	
ICU/HDU admission	1548 (95.7)	No	764 (81.4)	536 (78.9)	<0.001
		Yes	112 (11.9)	136 (20.0)	
		(Missing)	62 (6.6)	7 (1.0)	
Inotrope	1451 (89.7)	No	764 (81.4)	612 (90.1)	0.274
		Yes	47 (5.0)	28 (4.1)	
		(Missing)	127 (13.5)	39 (5.7)	
Total PEWS	1518 (93.9)	Median (IQR)	2.0 (1.0–4.0)	2.0 (1.0–5.0)	0.014
PEWS over 2	1518 (93.9)	No	471 (50.2)	351 (51.7)	0.315
		Yes	380 (40.5)	316 (46.5)	
		(Missing)	87 (9.3)	12 (1.8)	
Length of stay	1368 (84.6)	Median (IQR)	2.0 (1.0–3.0)	3.0 (1.0–7.0)	<0.001

*ICU* intensive care unit, *HDU* high dependency unit, *PEWS* Paediatric Early Warning Score at presentation.

### Asymptomatic and incidental SARS-CoV-2

We observed a significant increase in the proportion of patients who were asymptomatic at the time of SARS-CoV-2 detection from 10.4% (78/751) in the first wave to 24.7% (300/1214, *p* < 0.001) in the second wave (Supp Fig. E). CYP with asymptomatic or incidental SARS-CoV-2 were older (median age 11.2 years (IQR 1.5–15.9) vs 5.3 years (IQR 0.4–14.2, *p* < 0.001, Supp Table L), more likely to have hospital-acquired SARS-CoV-2 infection (12.4% (53/427) vs 4.2% (65/1540), *p* < 0.001), have a reported comorbidity (51.3% (219/427) vs 43.7% (673/1540), *p* = 0.006) and evidence of an alternative reason for admission (see *Supp Methods*, 30.9% (132/427) vs 5.3% (82/1540, *p* < 0.001). They also had a lower median PEWS on presentation (1.0 (IQR 0.0–2.0) vs 2.0 (IQR 1.0–4.0), *p* < 0.001). No differences were seen in sex or ethnicity or IMD.

## Discussion

The ISARIC WHO CCP-UK study recruited 2044 CYP with SARS-CoV-2 between 17th January 2020 and 31st January 2021 of whom 20.6% (427/2044) had asymptomatic or incidental SARS-CoV-2 infections. Of the symptomatic CYP, 5.6% (91/1617) had MIS-C and 16.0% (248/1548) were admitted to critical care. Within the symptomatic group, 0.8% (12/1504) died. There were 665 symptomatic CYP in W1 and 952 in W2, with those in W2 being older, more likely to be of South Asian ethnicity and with a lower proportion of hospital acquired SARS-CoV-2. Reassuringly, there was no evidence of a negative impact of relaxation in guidance to ‘shield’ vulnerable CYP in the latter part of 2020,^18^ with similar prevalence of comorbidities across the two waves.

Despite concerns about more severe disease and fatalities associated with the alpha variant in adults, relaxation of shielding advice and increases in face-to-face schooling, no difference was found in the proportion of symptomatic CYP admitted to critical care between the two waves, instead pointing to less severe disease in W2. After excluding patients with MIS-C, CYP admitted during W2 had a lower PEWS at presentation, lower antibiotic use and less respiratory and cardiovascular support compared to W1. Oral steroid use was higher in W2, likely because of changes in national guidance adopting the results of the RECOVERY trial.^19^ Whilst there was no difference in PEWS at presentation, respiratory or cardiovascular support in CYP with MIS-C between the waves, those in W2 were less likely to receive IVIg. This may reflect increases in clinician confidence, local guidance, or decreased availability of the therapy.

After exclusion of CYP with MIS-C, factors associated with admission to critical care remained very similar to our first report,^2^ with neonates and ages 10–14 and 15–19 years associated with admission in addition to ethnicity, PEWS at presentation and number of comorbidities.

Of particular interest were CYP without comorbidities. Those under 1 year comprised 42.4% of CYP admitted without comorbidities - an age group commonly admitted for brief periods of observation for viral illnesses. CYP with no reported comorbidities had a lower PEWS at presentation, shorter length of stay and received less respiratory support. However, 12.9% (112/876) of all hospitalised CYP without comorbidities were admitted to critical care, with 53 of these having MIS-C. CYP with MIS-C were also responsible for much of the invasive and non-invasive ventilation, inotrope use and IV steroids in the CYP group without comorbidities. Overall, the majority of children in hospital with symptomatic infection had no reported comorbidities (58.0% (938/1617). Of these CYP without reported comorbidities, 70.4% (660/938) were ≤ 11 years, representing a significant group in whom there is no current licenced vaccine available, while 47.5% (278/585) of CYP of vaccine-licensed age had no reported comorbidities. This suggests that targeting prevention strategies, such as vaccines, on the basis of comorbid status or risk groups will have somewhat limited impact on hospitalisation in CYP of vaccine licensed age and overall.

The ISARIC WHO CCP-UK study provides an extensive, detailed and prospective dataset, continuously collected since the start of the pandemic and is uniquely placed to monitor changes in the characteristics of hospitalised CYP with SARS-CoV-2 in the UK from emergence to evolution of the pandemic. Unlike routine and population-based studies, data collection for ISARIC is focused on characterisation of novel emerging disease. Due to the need to retain a ‘lite’ data collection process, some data terms (particularly comorbidities) are only relevant to adults and not relevant to CYP. Reporting to ISARIC is voluntary and is likely to lead an underestimate in the number of asymptomatic or incidental cases reported, as some sites may have chosen only to report ‘true’ COVID-19 cases. Genotype data was not available.

It is important to recall that the study definition of critical care is not just intensive care but includes high dependency units in secondary care centres. In paediatrics, patients are often admitted to these wards for close observation, without intensive therapy. This is borne out by our study finding much lower rates of high-level respiratory or cardiovascular support than rates of critical care admission.

Curtailment of analysis on 31st January 2021, after the peak of the second wave and with community cases falling, was planned due to the urgent need to provide paediatric data on the alpha variant to inform public health policy at that time. However, MIS-C typically presents 2-4 weeks after infection, and therefore cases of MIS-C due to infection acquired in the second wave will be underestimated.

A major strength of our study is that it highlights the importance of differentiating whether CYP are hospitalised because of COVID-19 i.e., disease caused by SARS-CoV-2 infection or with SARS-CoV-2 infection that was incidental. Analyses performed without excluding CYP where SARS-CoV-2 is incidental will produce misleading results. Describing and identifying factors predicting severity of COVID-19 could be biased in either direction by inclusion of both a high proportion of asymptomatic, or incidental infections, and inclusion of CYP admitted to critical care for unrelated reasons such as trauma.

Data collection before and after the emergence of the alpha variant provides reassuring evidence that clinical characteristics in CYP did not change over time coincident with the rise to dominance of this strain. There is evidence in adults that the alpha variant is not only associated with higher transmissibility, but also higher risk of hospital admission^20^ and death,^13,21,22^ although estimates of case-fatality rates may be limited by confounding factors.^21^ However, most studies did not include CYP, and reported risks appear to be age dependent. In a large community-based UK retrospective cohort, Nyberg et al. found an increased risk of hospital admission and mortality in adults older than 30 years testing positive for the alpha variant, but found no difference for young people under 20 years.^20^ In a brief report, Brookman et al. compared characteristics of 20 CYP admitted to a single London hospital in W1 to 60 CYP in W2 with no difference in demographics or increase in severity of disease.^23^ Our larger and more detailed study supports these findings.

Much of the focus of recent reports in SARS-CoV-2 in CYP aims to identify comorbidities associated with critical care admission,^24,25^ particularly in the discussion about vaccination of CYP. The granularity of our study allowed us to also examine CYP admitted to hospital without comorbidities in detail. While this group appears to be driven by infants with short hospital stays for brief observation, 12.9% of CYP admitted without comorbidities required critical care admission. CYP with MIS-C made up half of this group, but this also suggests that there may still be a group of previously well CYP with as yet unclear risk factors for critical care.

Understanding of asymptomatic SARS-CoV-2 infection has increased throughout the pandemic, particularly in CYP and it is clear that to be clinically useful, studies must identify CYP who have asymptomatic or incidental infection among those who are hospitalised. In our study, we found an increase in asymptomatic and incidental SARS-CoV-2 infections in W2 when compared to W1. Factors influencing this finding will include the introduction of routine testing of all hospital admissions and twice weekly lateral flow antigen testing in secondary schools (CYP ≥ 11 years). Two single-centre studies from the USA have reported that between 40 and 45% of their paediatric admissions with SARS-CoV-2 were either incidental or unlikely to be due to the virus itself.^26,27^ Brookman et al. reported a prevalence of asymptomatic/incidental infection in their cohort of 33%.^23^ Our finding that at least 21% of reported cases in our cohort were asymptomatic/incidental may be an underestimate given that reporting was voluntary and the variable use of free text to record these details.

Differences in demographics and symptomatology between W1 and W2 may reflect changes in testing patterns, infection control and surveillance practices over the course of the pandemic.^28^ UK hospitals gradually moved from testing based on case definition (with key symptoms of fever, cough, respiratory distress, or loss of sense of taste/smell) to universal testing of all admissions. Reduction in hospital-acquired infection may be due to improved infection control procedures, earlier detection of community acquired infection, or both.

Accepting the limitations above, we provide evidence suggesting the emergence of the alpha variant did not lead to more severe disease in CYP in the UK. With the Delta variant now dominating in the UK, our study serves an exemplar of both the strengths and limitations of large hospital-based studies in informing immediate public health approaches to emerging new variants. The key strength of our study is in providing a granularity of individual patient data which allows us to look in detail at clinical presentations and outcomes, identify important sources of bias, and provide comprehensive data over time. The key limitation is that this nuanced approach takes time to perform and is outpaced by the rapid evolution of this pandemic. As a result, initial incomplete data is by necessity used to inform policy, while more accurate information may only be gained in retrospect.

We urge other paediatric cohort studies to develop processes to define and record asymptomatic and incidental SARS-CoV-2 infection and differentiate this in analyses from COVID-19 disease. In addition, this study raises the possibility of as yet unidentified risk factors for critical care in CYP without comorbidities. As new variants of SARS-CoV-2 emerge, there is no guarantee that the generally mild disease observed in CYP to date will continue to predominate. Paediatricians and epidemiologists must remain vigilant in monitoring patterns of SARS-Cov-2 infection in CYP and develop more efficient systems to inform policy and clinical practice with speed and accuracy.

## Supplementary information

Supplementary Table

Supplementary Information

## Data Availability

Data are available for reuse through a secure data sharing platform. Access is welcome through the ISARIC4C Independent Data and Material Access Committee (https://isaric4c.net).
