# Supplementary material for: Comparison of UK paediatric SARS-CoV-2 admissions across the first and second pandemic waves
Source: Pediatr Res. 2022 Apr 22;93(1):207–16. doi: 10.1038/s41390-022-02052-5 (PMC9876790; doi:10.1038/s41390-022-02052-5)
Supplement: Supplementary file 2 — Supplementary Information [file 41390_2022_2052_MOESM2_ESM.docx]

# Supplementary Information

## Supplementary Methods

Data on clinical progress were collected on days 1, 3, 6 and 9 of admission and, if applicable, on the day of admission to critical care, as were data on interim outcome status at day 28 and final outcome (discharged alive/palliative discharge/in-hospital death) when that occured.

### Variables

#### Baseline vital signs were used to calculate a Paediatric Early Warning Score (PEWS) which provided a measure of severity of illness at presentation.^1^ In addition to the variables used for comorbidities in our original report, we also included a neurodevelopmental comorbidity category which included children and young people (CYP) with learning disability, autism spectrum disorders and attention deficit hyperactivity disorder.^2^ Oxygen delivered by high-flow nasal cannulae was assigned as “high flow support” which is available in critical care environments and on some wards. CYP admitted for >5 days before testing for SARS-CoV-2 infection were categorised as potential hospital acquired infection.

#### Length of stay

Length of stay was calculated from date of assessment in hospital for SARS-CoV-2 infection and date of discharge (where this was recorded) and was available for patients who had a recorded discharge by 28 days.

#### Age

Age was calculated based on date of birth and date of assessment in hospital for SARS-CoV-2 infection.

#### Indices of multiple deprivation (IMD)

IMD scores were derived from postal codes for usual residence transcribed from hospital records. IMD quintile 1 represents the most deprived and quintile 5 the least deprived.

#### Critical care

Paediatric intensive care units (PICUs) are dedicated care settings providing the highest level of critical care for children and young people, who usually need invasive mechanical ventilation or support for two or more organ systems with a higher nurse to patient ratio. PICUs are usually located in regional tertiary centres or specialised hospitals. Paediatric high dependency units (HDUs) are for patients needing close monitoring and therapies for single organ system support, usually without invasive ventilation. HDUs are provided at tertiary hospitals and most district general hospitals.

#### Duplicates

Each child is represented once in the dataset. In cases where the child was readmitted, the admission with the highest level of care was retained. If readmissions required the same level of care, the earliest admission was retained. Where the child was transferred from one participating site to another during the same episode of care, their data were considerred as one admission, retaining the first available vital signs and laboratory results and recording the highest level of treatments they had received.

#### Criteria for diagnosis of MIS-C

The case report form contained a Y/N variable for multisystem inflammatory syndrome in children (MIS-C) also known as paediatric inflammatory multisystem syndrome temporally associated with SARS-CoV-2 (PIMS-TS). We also adjusted the World Health Organisation (WHO) preliminary case definition for MIS-C, as previously described^2^ and searched free text in case report forms for “MIS-C”, “PIMS-TS” and “IVIg” (intravenous immunoglobulin). Patients identified using these three approaches were collated and sites contacted directly to clarify the diagnosis and to collect further details. Patients with pathogenic bacteria identified in blood or cerebrospinal fluid cultures and those with a diagnosis of appendicitis were censored from the MIS-C subgroup.

#### Incidental SARS-CoV-2

Coded “yes”:

- Patients where SARS-CoV-2 was noted to be incidental in free text.
- Patients with alternative reasons for admission, where free text notes no SARS-CoV-2 symptoms or asymptomatic.
- Patients where there is a clear primary reason for admission unrelated to any infection symptoms in free text e.g. overdose, road traffic accident.
- Patients where there is a clear primary reason for admission which relates to a specific focal infection not thought to be associated with SARS-CoV-2 e.g. septic arthritis, eczema herpeticum.

Coded “no / unknown”:

- No mention of other reason for admission in free text, no mention of asymptomatic or incidental finding
- Patient came to hospital for another reason (eg pre admission screen) but had SARS-CoV-2 symptoms and was admitted for this

Patients who had been coded “yes” for other reason for admission, but where it was not clear whether SARS-CoV-2 symptoms also contributed to presentation. For example:

- Appendicitis.
- Diabetic ketoacidosis.
- Systemic infection related admissions, where co-infection could be present.

#### Other reason for admission

**Coded “yes”**

Patients where free text includes reference to another acute condition which could have contributed to admission. This includes but is not limited to:

- Fractures, burns and other injuries.
- Eating disorder, self-harm, drug overdose, psychosis (note where free text states only depression or anxiety this was considered chronic, and not an alternative reason for admission unless stated otherwise).
- Surgical admissions including appendicitis.
- New presentation of type 1 diabetes.
- Neonatal jaundice.
- Patients admitted in labour or for elective caesarean section.

Patients where free text includes reference of admission related to a chronic condition. This includes, but is not limited to:

- Exacerbation of inflammatory bowel syndrome.
- Diabetic ketoacidosis in known diabetic.
- Elective admissions for diagnostic investigations.
- Elective admissions for surgical procedures or chemotherapy.

**Coded “no / unknown”**

Patients where free text includes reference to acute symptoms/presentation which could also represent symptoms of SARS-CoV-2. Examples:

- Febrile convulsions.
- Seizures in known epilepsy.
- Acute exacerbation of asthma.
- Gastroenteritis.

#### Missing data

Capacity to enrol into the current study was limited by staff availability, especially during admission surges research staff were redeployed to clinical activities. We did not impute missing data. All patients were admitted at least two weeks prior to the date of data extraction to minimise missing data. Denominators differ between analyses owing to incomplete data recorded for some variables. The research team undertook data cleaning and source verification to ensure the data extracted and analysed were as accurate as possible.

## Supplementary Results­­

#### Assessment of potential sources of bias

In total, 764 CYP were admitted during the first wave (17^th^ January to 31st July 2020) and 1,280 during the second wave (1^st^ August 2020 to 31st January 2021) across a total of 187 sites of which 23 had access to an onsite paediatric intensive care unit (PICU) (*Supplementary Figure C* and *Supplementary Table A*). As reporting to ISARIC is not mandatory, we examined whether sites with onsite PICUs reported more CYP in one wave than another, which might bias the severity of illness in reported patients. 118 hospitals reported paediatric patients to ISARIC in both the first and second waves, 34 more reported in the first wave only and 35 in the second wave only. The proportion of patients reported from hospitals with access to an onsite PICU did not differ between the waves (37.0% (283/764) in the first wave vs 35.5% (455/1280, p = 0.53) in the second wave).

We then compared the number of CYP reported to ISARIC against the numbers of local SARS-CoV-2 cases identified by Pillar 1 and 2 testing by Public Health England across NHS regions (*Supplementary Figure D*). In the ISARIC dataset, regional peaks were seen in the Midlands in November 2020 and in London in December 2020 which closely mirrored those reported by Public Health England at the same time points, indicating that ISARIC also captured localised SARS-CoV-2 peaks across the UK.^3^

# Supplementary Tables

| Wave | Hospitals | Patients | Onsite PICU |
| --- | --- | --- | --- |
| First only | 34 | 99 | 2 |
| First and Second | 118 | 1747 | 20 |
| Second only | 35 | 198 | 1 |

**Supplementary Table A**. Comparison of the number of sites reporting to ISARIC in the first and second waves against the number of patients reporting and whether the hospital had an on-site PICU.

|  |  | First | Second | p |
| --- | --- | --- | --- | --- |
| Total N (%) |  | 665 (41.1) | 952 (58.9) |  |
| History of fever | No | 148 (22.3) | 311 (32.7) | <0.001 |
|  | Yes | 491 (73.8) | 544 (57.1) |  |
|  | (Missing) | 26 (3.9) | 97 (10.2) |  |
| Cough | No | 353 (53.1) | 519 (54.5) | 0.056 |
|  | Yes | 269 (40.5) | 320 (33.6) |  |
|  | (Missing) | 43 (6.5) | 113 (11.9) |  |
| Cough: with sputum production | No | 486 (73.1) | 663 (69.6) | 0.516 |
|  | Yes | 40 (6.0) | 46 (4.8) |  |
|  | (Missing) | 139 (20.9) | 243 (25.5) |  |
| Sore throat | No | 398 (59.8) | 558 (58.6) | 0.183 |
|  | Yes | 59 (8.9) | 106 (11.1) |  |
|  | (Missing) | 208 (31.3) | 288 (30.3) |  |
| Runny nose (Rhinorrhoea) | No | 438 (65.9) | 616 (64.7) | 0.423 |
|  | Yes | 84 (12.6) | 135 (14.2) |  |
|  | (Missing) | 143 (21.5) | 201 (21.1) |  |
| Ear pain (Otalgia) | No | 433 (65.1) | 634 (66.6) | 0.809 |
|  | Yes | 11 (1.7) | 19 (2.0) |  |
|  | (Missing) | 221 (33.2) | 299 (31.4) |  |
| Wheezing | No | 508 (76.4) | 712 (74.8) | 0.607 |
|  | Yes | 53 (8.0) | 66 (6.9) |  |
|  | (Missing) | 104 (15.6) | 174 (18.3) |  |
| Muscle aches (Myalgia) | No | 390 (58.6) | 586 (61.6) | 0.337 |
|  | Yes | 51 (7.7) | 62 (6.5) |  |
|  | (Missing) | 224 (33.7) | 304 (31.9) |  |
| Joint pain (Arthralgia) | No | 420 (63.2) | 609 (64.0) | 0.213 |
|  | Yes | 18 (2.7) | 39 (4.1) |  |
|  | (Missing) | 227 (34.1) | 304 (31.9) |  |
| Fatigue / Malaise | No | 331 (49.8) | 496 (52.1) | 0.471 |
|  | Yes | 162 (24.4) | 220 (23.1) |  |
|  | (Missing) | 172 (25.9) | 236 (24.8) |  |
| Shortness of breath (Dyspnea) | No | 406 (61.1) | 574 (60.3) | 0.174 |
|  | Yes | 185 (27.8) | 221 (23.2) |  |
|  | (Missing) | 74 (11.1) | 157 (16.5) |  |
| Lower chest wall indrawing | No | 475 (71.4) | 699 (73.4) | 0.196 |
|  | Yes | 41 (6.2) | 44 (4.6) |  |
|  | (Missing) | 149 (22.4) | 209 (22.0) |  |
| Headache | No | 375 (56.4) | 544 (57.1) | 0.690 |
|  | Yes | 66 (9.9) | 104 (10.9) |  |
|  | (Missing) | 224 (33.7) | 304 (31.9) |  |
| Altered consciousness / confusion | No | 516 (77.6) | 716 (75.2) | 0.803 |
|  | Yes | 42 (6.3) | 54 (5.7) |  |
|  | (Missing) | 107 (16.1) | 182 (19.1) |  |
| Seizures | No | 539 (81.1) | 729 (76.6) | 0.951 |
|  | Yes | 37 (5.6) | 52 (5.5) |  |
|  | (Missing) | 89 (13.4) | 171 (18.0) |  |
| Abdominal pain | No | 363 (54.6) | 544 (57.1) | 0.219 |
|  | Yes | 126 (18.9) | 158 (16.6) |  |
|  | (Missing) | 176 (26.5) | 250 (26.3) |  |
| Vomiting / Nausea | No | 377 (56.7) | 553 (58.1) | 0.336 |
|  | Yes | 206 (31.0) | 269 (28.3) |  |
|  | (Missing) | 82 (12.3) | 130 (13.7) |  |
| Diarrhoea | No | 467 (70.2) | 666 (70.0) | 0.374 |
|  | Yes | 107 (16.1) | 133 (14.0) |  |
|  | (Missing) | 91 (13.7) | 153 (16.1) |  |
| Conjunctivitis | No | 498 (74.9) | 703 (73.8) | 0.581 |
|  | Yes | 28 (4.2) | 33 (3.5) |  |
|  | (Missing) | 139 (20.9) | 216 (22.7) |  |
| Skin rash | No | 481 (72.3) | 693 (72.8) | 0.096 |
|  | Yes | 96 (14.4) | 106 (11.1) |  |
|  | (Missing) | 88 (13.2) | 153 (16.1) |  |
| Lymphadenopathy | No | 490 (73.7) | 721 (75.7) | 0.009 |
|  | Yes | 27 (4.1) | 17 (1.8) |  |
|  | (Missing) | 148 (22.3) | 214 (22.5) |  |
| Bleeding (Haemorrhage) | No | 542 (81.5) | 775 (81.4) | 0.091 |
|  | Yes | 14 (2.1) | 9 (0.9) |  |
|  | (Missing) | 109 (16.4) | 168 (17.6) |  |

**Supplementary Table B.** Presenting symptoms by wave, with CYP with asymptomatic / incidental SARS-CoV-2 infections excluded.

|  |  | First | Second | p |
| --- | --- | --- | --- | --- |
| Total N (%) |  | 665 (41.1) | 952 (58.9) |  |
| Any comorbidity | No/Unknown | 367 (55.2) | 571 (60.0) | 0.062 |
|  | Yes | 298 (44.8) | 381 (40.0) |  |
| Prematurity | No | 160 (24.1) | 206 (21.6) | 0.159 |
|  | Yes | 43 (6.5) | 38 (4.0) |  |
|  | (Missing) | 462 (69.5) | 708 (74.4) |  |
| Neurological | No | 570 (85.7) | 770 (80.9) | 0.526 |
|  | Yes | 67 (10.1) | 102 (10.7) |  |
|  | (Missing) | 28 (4.2) | 80 (8.4) |  |
| Neurodisability | No | 577 (86.8) | 785 (82.5) | 0.900 |
|  | Yes | 32 (4.8) | 41 (4.3) |  |
|  | (Missing) | 56 (8.4) | 126 (13.2) |  |
| Neurodevelopmental | No | 590 (88.7) | 773 (81.2) | 0.648 |
|  | Yes | 27 (4.1) | 41 (4.3) |  |
|  | (Missing) | 48 (7.2) | 138 (14.5) |  |
| Respiratory | No | 599 (90.1) | 832 (87.4) | 0.378 |
|  | Yes | 34 (5.1) | 37 (3.9) |  |
|  | (Missing) | 32 (4.8) | 83 (8.7) |  |
| Asthma | No | 590 (88.7) | 787 (82.7) | 0.149 |
|  | Yes | 49 (7.4) | 87 (9.1) |  |
|  | (Missing) | 26 (3.9) | 78 (8.2) |  |
| Cardiac | No | 597 (89.8) | 835 (87.7) | 0.048 |
|  | Yes | 40 (6.0) | 34 (3.6) |  |
|  | (Missing) | 28 (4.2) | 83 (8.7) |  |
| Gastrointestinal | No | 614 (92.3) | 847 (89.0) | 0.444 |
|  | Yes | 22 (3.3) | 23 (2.4) |  |
|  | (Missing) | 29 (4.4) | 82 (8.6) |  |
| Haematology / Oncology / Immunology | No | 588 (88.4) | 815 (85.6) | 0.373 |
|  | Yes | 47 (7.1) | 53 (5.6) |  |
|  | (Missing) | 30 (4.5) | 84 (8.8) |  |
| Obesity | No | 607 (91.3) | 808 (84.9) | 0.949 |
|  | Yes | 19 (2.9) | 27 (2.8) |  |
|  | (Missing) | 39 (5.9) | 117 (12.3) |  |
| Malnutrition | No | 626 (94.1) | 835 (87.7) | 0.169 |
|  | Yes | 5 (0.8) | 15 (1.6) |  |
|  | (Missing) | 34 (5.1) | 102 (10.7) |  |
| Diabetes | No | 621 (93.4) | 844 (88.7) | 0.477 |
|  | Yes | 17 (2.6) | 17 (1.8) |  |
|  | (Missing) | 27 (4.1) | 91 (9.6) |  |
| Other endocrine | No | 607 (91.3) | 805 (84.6) | 0.542 |
|  | Yes | 10 (1.5) | 9 (0.9) |  |
|  | (Missing) | 48 (7.2) | 138 (14.5) |  |
| Genetic | No | 593 (89.2) | 781 (82.0) | 0.983 |
|  | Yes | 24 (3.6) | 33 (3.5) |  |
|  | (Missing) | 48 (7.2) | 138 (14.5) |  |
| Renal | No | 623 (93.7) | 846 (88.9) | 0.603 |
|  | Yes | 14 (2.1) | 24 (2.5) |  |
|  | (Missing) | 28 (4.2) | 82 (8.6) |  |
| Metabolic | No | 615 (92.5) | 807 (84.8) | 0.351 |
|  | Yes | 2 (0.3) | 7 (0.7) |  |
|  | (Missing) | 48 (7.2) | 138 (14.5) |  |
| Rheumatology | No | 631 (94.9) | 858 (90.1) | 0.182 |
|  | Yes | 5 (0.8) | 15 (1.6) |  |
|  | (Missing) | 29 (4.4) | 79 (8.3) |  |
| Other | No | 576 (86.6) | 772 (81.1) | 0.274 |
|  | Yes | 42 (6.3) | 43 (4.5) |  |
|  | (Missing) | 47 (7.1) | 137 (14.4) |  |

**Supplementary Table C.** Comparison of comorbidities across the two waves, CYP with asymptomatic or incidental SARS-CoV-2 excluded.

|  |  | First | Second | p |
| --- | --- | --- | --- | --- |
| Total N (%) |  | 764 (37.4) | 1280 (62.6) |  |
| Any comorbidity | No/Unknown | 408 (53.4) | 738 (57.7) | 0.065 |
|  | Yes | 356 (46.6) | 542 (42.3) |  |
| Prematurity | No | 175 (22.9) | 229 (17.9) | 0.173 |
|  | Yes | 49 (6.4) | 47 (3.7) |  |
|  | (Missing) | 540 (70.7) | 1004 (78.4) |  |
| Neurological | No | 652 (85.3) | 1066 (83.3) | 0.939 |
|  | Yes | 78 (10.2) | 126 (9.8) |  |
|  | (Missing) | 34 (4.5) | 88 (6.9) |  |
| Neurodisability | No | 660 (86.4) | 1084 (84.7) | 0.162 |
|  | Yes | 37 (4.8) | 44 (3.4) |  |
|  | (Missing) | 67 (8.8) | 152 (11.9) |  |
| Neurodevelopmental | No | 674 (88.2) | 1011 (79.0) | 1.000 |
|  | Yes | 33 (4.3) | 49 (3.8) |  |
|  | (Missing) | 57 (7.5) | 220 (17.2) |  |
| Respiratory | No | 689 (90.2) | 1146 (89.5) | 0.101 |
|  | Yes | 38 (5.0) | 43 (3.4) |  |
|  | (Missing) | 37 (4.8) | 91 (7.1) |  |
| Asthma | No | 679 (88.9) | 1090 (85.2) | 0.266 |
|  | Yes | 53 (6.9) | 104 (8.1) |  |
|  | (Missing) | 32 (4.2) | 86 (6.7) |  |
| Cardiac | No | 686 (89.8) | 1141 (89.1) | 0.046 |
|  | Yes | 44 (5.8) | 47 (3.7) |  |
|  | (Missing) | 34 (4.5) | 92 (7.2) |  |
| Gastrointestinal | No | 706 (92.4) | 1160 (90.6) | 0.323 |
|  | Yes | 24 (3.1) | 30 (2.3) |  |
|  | (Missing) | 34 (4.5) | 90 (7.0) |  |
| Haematology / Oncology / Immunology | No | 676 (88.5) | 1116 (87.2) | 0.296 |
|  | Yes | 53 (6.9) | 72 (5.6) |  |
|  | (Missing) | 35 (4.6) | 92 (7.2) |  |
| Obesity | No | 698 (91.4) | 1112 (86.9) | 0.781 |
|  | Yes | 20 (2.6) | 36 (2.8) |  |
|  | (Missing) | 46 (6.0) | 132 (10.3) |  |
| Malnutrition | No | 715 (93.6) | 1145 (89.5) | 0.092 |
|  | Yes | 7 (0.9) | 24 (1.9) |  |
|  | (Missing) | 42 (5.5) | 111 (8.7) |  |
| Diabetes | No | 714 (93.5) | 1158 (90.5) | 0.413 |
|  | Yes | 18 (2.4) | 22 (1.7) |  |
|  | (Missing) | 32 (4.2) | 100 (7.8) |  |
| Other endocrine | No | 697 (91.2) | 1050 (82.0) | 0.368 |
|  | Yes | 10 (1.3) | 10 (0.8) |  |
|  | (Missing) | 57 (7.5) | 220 (17.2) |  |
| Genetic | No | 680 (89.0) | 1022 (79.8) | 0.798 |
|  | Yes | 27 (3.5) | 38 (3.0) |  |
|  | (Missing) | 57 (7.5) | 220 (17.2) |  |
| Renal | No | 712 (93.2) | 1162 (90.8) | 0.762 |
|  | Yes | 19 (2.5) | 28 (2.2) |  |
|  | (Missing) | 33 (4.3) | 90 (7.0) |  |
| Metabolic | No | 705 (92.3) | 1053 (82.3) | 0.330 |
|  | Yes | 2 (0.3) | 7 (0.5) |  |
|  | (Missing) | 57 (7.5) | 220 (17.2) |  |
| Rheumatology | No | 724 (94.8) | 1176 (91.9) | 0.211 |
|  | Yes | 6 (0.8) | 18 (1.4) |  |
|  | (Missing) | 34 (4.5) | 86 (6.7) |  |
| Other | No | 637 (83.4) | 938 (73.3) | 0.319 |
|  | Yes | 73 (9.6) | 127 (9.9) |  |
|  | (Missing) | 54 (7.1) | 215 (16.8) |  |

**Supplementary Table D.** Comparison of comorbidities for the whole cohort (i.e. includes asymptomatic or incidental SARS-CoV-2) across the two waves.

|  | Total N |  | First | Second | p |
| --- | --- | --- | --- | --- | --- |
| Total N (%) |  |  | 764 (37.4) | 1280 (62.6) |  |
| Antibiotic medication | 1883 (92.1) | No | 231 (30.2) | 557 (43.5) | <0.001 |
|  |  | Yes | 502 (65.7) | 593 (46.3) |  |
|  |  | (Missing) | 31 (4.1) | 130 (10.2) |  |
| Antiviral | 1885 (92.2) | No | 680 (89.0) | 1100 (85.9) | 0.215 |
|  |  | Yes | 47 (6.2) | 58 (4.5) |  |
|  |  | (Missing) | 37 (4.8) | 122 (9.5) |  |
| Maximal steroid therapy | 1820 (89.0) | None | 598 (78.3) | 955 (74.6) | <0.001 |
|  |  | Oral | 38 (5.0) | 144 (11.2) |  |
|  |  | IV | 45 (5.9) | 40 (3.1) |  |
|  |  | (Missing) | 83 (10.9) | 141 (11.0) |  |
| Maximum respiratory support | 1974 (96.6) | No respiratory support | 551 (72.1) | 979 (76.5) | <0.001 |
|  |  | Supplemental oxygen | 69 (9.0) | 123 (9.6) |  |
|  |  | High flow support | 36 (4.7) | 37 (2.9) |  |
|  |  | Non-invasive | 38 (5.0) | 31 (2.4) |  |
|  |  | Invasive | 59 (7.7) | 51 (4.0) |  |
|  |  | (Missing) | 11 (1.4) | 59 (4.6) |  |
| ICU/HDU admission | 1972 (96.5) | No | 620 (81.2) | 1007 (78.7) | 1.000 |
|  |  | Yes | 132 (17.3) | 213 (16.6) |  |
|  |  | (Missing) | 12 (1.6) | 60 (4.7) |  |
| Inotrope | 1856 (90.8) | No | 673 (88.1) | 1102 (86.1) | <0.001 |
|  |  | Yes | 52 (6.8) | 29 (2.3) |  |
|  |  | (Missing) | 39 (5.1) | 149 (11.6) |  |
| Total PEWS | 1920 (93.9) | Median (IQR) | 2.0 (1.0 to 4.0) | 2.0 (1.0 to 4.0) | <0.001 |
| PEWS over 2 | 1920 (93.9) | No | 391 (51.2) | 751 (58.7) | <0.001 |
|  |  | Yes | 349 (45.7) | 429 (33.5) |  |
|  |  | (Missing) | 24 (3.1) | 100 (7.8) |  |
| Length of stay | 1727 (84.5) | Median (IQR) | 2.0 (1.0 to 6.0) | 2.0 (1.0 to 4.0) | <0.001 |

**Supplementary Table E.** Comparison of treatments received by children by wave across the whole cohort (i.e. includes asymptomatic and incidental SARS-CoV-2). ICU = intensive care unit, HDU = high dependency unit. PEWS = Paediatric Early Warning Score at presentation.

|  | Total N |  | First | Second | p |
| --- | --- | --- | --- | --- | --- |
| Total N (%) |  |  | 49 (53.8) | 42 (46.2) |  |
| Antibiotic medication | 83 (91.2) | No | 4 (8.2) | 1 (2.4) | 0.644 |
|  |  | Yes | 45 (91.8) | 33 (78.6) |  |
|  |  | (Missing) | 0 (0.0) | 8 (19.0) |  |
| Maximal steroid therapy | 72 (79.1) | None | 12 (24.5) | 15 (35.7) | 0.200 |
|  |  | Oral | 2 (4.1) | 3 (7.1) |  |
|  |  | IV | 26 (53.1) | 14 (33.3) |  |
|  |  | (Missing) | 9 (18.4) | 10 (23.8) |  |
| IVIg | 91 (100.0) | No | 8 (16.3) | 17 (40.5) | 0.018 |
|  |  | Yes | 41 (83.7) | 25 (59.5) |  |
| Immunomodulator | 89 (97.8) | No | 41 (83.7) | 37 (88.1) | 0.537 |
|  |  | Yes | 7 (14.3) | 4 (9.5) |  |
|  |  | (Missing) | 1 (2.0) | 1 (2.4) |  |
| Maximum respiratory support | 91 (100.0) | No respiratory support | 22 (44.9) | 24 (57.1) | 0.458 |
|  |  | Supplemental oxygen | 3 (6.1) | 5 (11.9) |  |
|  |  | High flow support | 4 (8.2) | 2 (4.8) |  |
|  |  | Non-invasive | 8 (16.3) | 3 (7.1) |  |
|  |  | Invasive | 12 (24.5) | 8 (19.0) |  |
| ICU/HDU admission | 89 (97.8) | No | 13 (26.5) | 15 (35.7) | 0.359 |
|  |  | Yes | 36 (73.5) | 25 (59.5) |  |
|  |  | (Missing) | 0 (0.0) | 2 (4.8) |  |
| Inotrope | 91 (100.0) | No | 23 (46.9) | 22 (52.4) | 0.676 |
|  |  | Yes | 26 (53.1) | 20 (47.6) |  |
| Total PEWS | 91 (100.0) | Median (IQR) | 5.0 (2.0 to 6.0) | 4.0 (2.0 to 6.0) | 0.273 |
| PEWS over 2 | 91 (100.0) | No | 14 (28.6) | 15 (35.7) | 0.505 |
|  |  | Yes | 35 (71.4) | 27 (64.3) |  |
| Length of stay | 67 (73.6) | Median (IQR) | 8.5 (5.8 to 12.0) | 6.0 (4.0 to 10.0) | 0.031 |

**Supplementary Table F.** Treatments received by children with MIS-C by wave. ICU = intensive care unit, HDU = high dependency unit, PEWS = Paediatric Early Warning Score at presentation, IQR = interquartile range.

|  | Total N |  | Ward-level care | Critical care | p |
| --- | --- | --- | --- | --- | --- |
| Total N (%) |  |  | 1300 (84.0) | 248 (16.0) |  |
| Age | 1548 (100.0) | <1 mth | 90 (6.9) | 23 (9.3) | <0.001 |
|  |  | >1mth <1 y | 372 (28.6) | 27 (10.9) |  |
|  |  | 1-4 y | 205 (15.8) | 41 (16.5) |  |
|  |  | 5-9 y | 145 (11.2) | 38 (15.3) |  |
|  |  | 10-14 y | 198 (15.2) | 68 (27.4) |  |
|  |  | 15-19 y | 290 (22.3) | 51 (20.6) |  |
| Licenced vaccine available | 1548 (100.0) | No (<= 11 years) | 847 (65.2) | 146 (58.9) | 0.069 |
|  |  | Yes (>= 12 years) | 453 (34.8) | 102 (41.1) |  |
| Sex at Birth | 1546 (99.9) | Male | 695 (53.5) | 138 (55.6) | 0.590 |
|  |  | Female | 603 (46.4) | 110 (44.4) |  |
|  |  | (Missing) | 2 (0.2) | 0 (0.0) |  |
| Ethnicity | 1341 (86.6) | White | 697 (53.6) | 85 (34.3) | <0.001 |
|  |  | Black | 74 (5.7) | 25 (10.1) |  |
|  |  | South Asian | 173 (13.3) | 47 (19.0) |  |
|  |  | Other ethnic minority | 184 (14.2) | 56 (22.6) |  |
|  |  | (Missing) | 172 (13.2) | 35 (14.1) |  |
| IMD quintile | 1432 (92.5) | 1 (most deprived) | 449 (34.5) | 72 (29.0) | 0.385 |
|  |  | 2 | 263 (20.2) | 35 (14.1) |  |
|  |  | 3 | 190 (14.6) | 30 (12.1) |  |
|  |  | 4 | 153 (11.8) | 34 (13.7) |  |
|  |  | 5 (least deprived) | 179 (13.8) | 27 (10.9) |  |
|  |  | (Missing) | 66 (5.1) | 50 (20.2) |  |
| Potential hospital acquired SARS-CoV-2 | 1548 (100.0) | No | 1256 (96.6) | 227 (91.5) | <0.001 |
|  |  | Yes | 44 (3.4) | 21 (8.5) |  |
| PEWS over 2 | 1511 (97.6) | No | 737 (56.7) | 81 (32.7) | <0.001 |
|  |  | Yes | 531 (40.8) | 162 (65.3) |  |
|  |  | (Missing) | 32 (2.5) | 5 (2.0) |  |
| Any comorbidity | 1548 (100.0) | No/Unknown | 764 (58.8) | 112 (45.2) | <0.001 |
|  |  | Yes | 536 (41.2) | 136 (54.8) |  |
| Comorbidity count | 1548 (100.0) | Median (IQR) | 0.0 (0.0 to 1.0) | 1.0 (0.0 to 1.0) | <0.001 |
| Length of stay | 1358 (87.7) | Median (IQR) | 2.0 (1.0 to 3.2) | 8.0 (4.0 to 12.0) | <0.001 |

**Supplementary Table G**. Demographics and key clinical characteristics of children stratified by critical care admission (excluding asymptomatic or incidental SARS-CoV-2 infections but including those with MIS-C). IMD = indices of multiple deprivtation. PEWS = Paediatric Early Warning Score at presentation.

|  |  | Ward-level care | Critical care | p |
| --- | --- | --- | --- | --- |
| Total N (%) |  | 1300 (84.0) | 248 (16.0) |  |
| Any comorbidity | No/Unknown | 764 (58.8) | 112 (45.2) | <0.001 |
|  | Yes | 536 (41.2) | 136 (54.8) |  |
| Prematurity | No | 343 (26.4) | 22 (8.9) | <0.001 |
|  | Yes | 57 (4.4) | 21 (8.5) |  |
|  | (Missing) | 900 (69.2) | 205 (82.7) |  |
| Neurological | No | 1136 (87.4) | 197 (79.4) | <0.001 |
|  | Yes | 121 (9.3) | 46 (18.5) |  |
|  | (Missing) | 43 (3.3) | 5 (2.0) |  |
| Neurodisability | No | 1151 (88.5) | 204 (82.3) | 0.008 |
|  | Yes | 53 (4.1) | 20 (8.1) |  |
|  | (Missing) | 96 (7.4) | 24 (9.7) |  |
| Neurodevelopmental | No | 1165 (89.6) | 190 (76.6) | 0.589 |
|  | Yes | 56 (4.3) | 11 (4.4) |  |
|  | (Missing) | 79 (6.1) | 47 (19.0) |  |
| Respiratory | No | 1203 (92.5) | 221 (89.1) | 0.003 |
|  | Yes | 49 (3.8) | 21 (8.5) |  |
|  | (Missing) | 48 (3.7) | 6 (2.4) |  |
| Asthma | No | 1149 (88.4) | 220 (88.7) | 0.467 |
|  | Yes | 111 (8.5) | 25 (10.1) |  |
|  | (Missing) | 40 (3.1) | 3 (1.2) |  |
| Cardiac | No | 1204 (92.6) | 221 (89.1) | 0.001 |
|  | Yes | 49 (3.8) | 23 (9.3) |  |
|  | (Missing) | 47 (3.6) | 4 (1.6) |  |
| Gastrointestinal | No | 1227 (94.4) | 227 (91.5) | 0.001 |
|  | Yes | 28 (2.2) | 16 (6.5) |  |
|  | (Missing) | 45 (3.5) | 5 (2.0) |  |
| Haematology / Oncology / Immunology | No | 1169 (89.9) | 226 (91.1) | 1.000 |
|  | Yes | 84 (6.5) | 16 (6.5) |  |
|  | (Missing) | 47 (3.6) | 6 (2.4) |  |
| Obesity | No | 1181 (90.8) | 226 (91.1) | 0.041 |
|  | Yes | 33 (2.5) | 13 (5.2) |  |
|  | (Missing) | 86 (6.6) | 9 (3.6) |  |
| Malnutrition | No | 1217 (93.6) | 237 (95.6) | 1.000 |
|  | Yes | 16 (1.2) | 3 (1.2) |  |
|  | (Missing) | 67 (5.2) | 8 (3.2) |  |
| Diabetes | No | 1222 (94.0) | 235 (94.8) | 0.813 |
|  | Yes | 28 (2.2) | 6 (2.4) |  |
|  | (Missing) | 50 (3.8) | 7 (2.8) |  |
| Other endocrine | No | 1207 (92.8) | 197 (79.4) | 0.306 |
|  | Yes | 14 (1.1) | 4 (1.6) |  |
|  | (Missing) | 79 (6.1) | 47 (19.0) |  |
| Genetic | No | 1175 (90.4) | 193 (77.8) | 0.843 |
|  | Yes | 46 (3.5) | 8 (3.2) |  |
|  | (Missing) | 79 (6.1) | 47 (19.0) |  |
| Renal | No | 1223 (94.1) | 240 (96.8) | 0.498 |
|  | Yes | 32 (2.5) | 4 (1.6) |  |
|  | (Missing) | 45 (3.5) | 4 (1.6) |  |
| Metabolic | No | 1212 (93.2) | 201 (81.0) | 0.623 |
|  | Yes | 9 (0.7) | 0 (0.0) |  |
|  | (Missing) | 79 (6.1) | 47 (19.0) |  |
| Rheumatology | No | 1247 (95.9) | 234 (94.4) | <0.001 |
|  | Yes | 10 (0.8) | 10 (4.0) |  |
|  | (Missing) | 43 (3.3) | 4 (1.6) |  |
| Other | No | 1145 (88.1) | 194 (78.2) | 0.146 |
|  | Yes | 78 (6.0) | 7 (2.8) |  |
|  | (Missing) | 77 (5.9) | 47 (19.0) |  |

**Supplementary Table H**. Comorbidities of CYP stratified by critical care admission excluding asymptomatic or incidental SARS-CoV-2 infections but including those with MIS-C.

|  |  | Ward-level care | Critical care | OR (univariable) | OR (multivariable) | OR (multilevel) |
| --- | --- | --- | --- | --- | --- | --- |
| Wave | First | 526 (87.1) | 78 (12.9) | - | - | - |
|  | Second | 746 (87.3) | 109 (12.7) | 0.99 (0.72-1.35, p=0.926) | 0.91 (0.61-1.39, p=0.674) | 0.95 (0.61-1.48, p=0.823) |
| Age | >1mth <1 y | 371 (93.2) | 27 (6.8) | - | - | - |
|  | <1 mth | 90 (79.6) | 23 (20.4) | 3.51 (1.91-6.41, p<0.001) | 8.14 (3.73-18.29, p<0.001) | 9.27 (4.03-21.31, p<0.001) |
|  | 1-4 y | 199 (84.7) | 36 (15.3) | 2.49 (1.47-4.25, p=0.001) | 2.38 (1.14-5.07, p=0.022) | 2.28 (1.05-4.93, p=0.036) |
|  | 5-9 y | 133 (88.1) | 18 (11.9) | 1.86 (0.98-3.46, p=0.053) | 1.46 (0.58-3.55, p=0.413) | 1.43 (0.56-3.63, p=0.456) |
|  | 10-14 y | 189 (83.6) | 37 (16.4) | 2.69 (1.60-4.59, p<0.001) | 2.97 (1.42-6.43, p=0.005) | 2.90 (1.33-6.32, p=0.007) |
|  | 15-19 y | 290 (86.3) | 46 (13.7) | 2.18 (1.33-3.63, p=0.002) | 2.51 (1.26-5.24, p=0.011) | 2.72 (1.29-5.71, p=0.008) |
| Sex at Birth | Male | 674 (86.9) | 102 (13.1) | - | - | - |
|  | Female | 596 (87.5) | 85 (12.5) | 0.94 (0.69-1.28, p=0.706) | 0.95 (0.63-1.44, p=0.811) | 0.99 (0.64-1.53, p=0.969) |
| Ethnicity | White | 683 (90.6) | 71 (9.4) | - | - | - |
|  | Black | 71 (82.6) | 15 (17.4) | 2.03 (1.07-3.65, p=0.022) | 1.05 (0.42-2.36, p=0.913) | 1.00 (0.40-2.47, p=0.996) |
|  | South Asian | 167 (81.1) | 39 (18.9) | 2.25 (1.46-3.42, p<0.001) | 1.17 (0.63-2.10, p=0.611) | 1.35 (0.70-2.60, p=0.367) |
|  | Other ethnic minority | 181 (82.3) | 39 (17.7) | 2.07 (1.35-3.15, p=0.001) | 2.53 (1.51-4.21, p<0.001) | 2.53 (1.47-4.35, p=0.001) |
| IMD quintile | 1 (most deprived) | 438 (89.9) | 49 (10.1) | - | - | - |
|  | 2 | 256 (90.8) | 26 (9.2) | 0.91 (0.54-1.48, p=0.705) | 0.97 (0.53-1.73, p=0.918) | 1.00 (0.54-1.86, p=0.993) |
|  | 3 | 187 (89.9) | 21 (10.1) | 1.00 (0.57-1.70, p=0.989) | 1.10 (0.58-2.02, p=0.763) | 1.24 (0.64-2.41, p=0.526) |
|  | 4 | 151 (86.8) | 23 (13.2) | 1.36 (0.79-2.29, p=0.253) | 1.68 (0.87-3.16, p=0.115) | 1.84 (0.92-3.71, p=0.087) |
|  | 5 (least deprived) | 175 (88.8) | 22 (11.2) | 1.12 (0.65-1.89, p=0.668) | 1.13 (0.57-2.16, p=0.728) | 1.30 (0.63-2.66, p=0.477) |
| Number of comorbidities | 0 | 744 (92.7) | 59 (7.3) | - | - | - |
|  | 1 | 336 (82.8) | 70 (17.2) | 2.63 (1.82-3.81, p<0.001) | 4.03 (2.45-6.76, p<0.001) | 3.87 (2.28-6.56, p<0.001) |
|  | 2+ | 192 (76.8) | 58 (23.2) | 3.81 (2.56-5.66, p<0.001) | 4.25 (2.40-7.56, p<0.001) | 4.04 (2.22-7.35, p<0.001) |
| PEWS over 2 | No | 725 (91.8) | 65 (8.2) | - | - | - |
|  | Yes | 515 (81.5) | 117 (18.5) | 2.53 (1.84-3.52, p<0.001) | 5.17 (3.29-8.36, p<0.001) | 5.11 (3.16-8.27, p<0.001) |

**Supplementary Table I.** *Multivariable analysis of factors associated with admission to critical care unit* (excluding asymptomatic and incidental SARS-CoV-2 infections, and patients with Multisystem inflammatory syndrome in children (MIS-C)). Row percentages. IMD = Index of multiple deprivation. PEWS = Paediatric Early Warning Score at presentation. Odds ratios (OR) and 95% confidence intervals are presented.

|  | Total N |  | No/Unknown  Comorbidity | Comorbidity  present | p |
| --- | --- | --- | --- | --- | --- |
| Total N (%) |  |  | 938 (58.0) | 679 (42.0) |  |
| Age at assessment (Years) | 1617 (100.0) | Median (IQR) | 2.0 (0.2 to 12.9) | 9.9 (1.9 to 15.3) | <0.001 |
| Age | 1617 (100.0) | <1 mth | 91 (9.7) | 31 (4.6) | <0.001 |
|  |  | >1mth <1 y | 307 (32.7) | 100 (14.7) |  |
|  |  | 1-4 y | 145 (15.5) | 111 (16.3) |  |
|  |  | 5-9 y | 89 (9.5) | 102 (15.0) |  |
|  |  | 10-14 y | 136 (14.5) | 148 (21.8) |  |
|  |  | 15-19 y | 170 (18.1) | 187 (27.5) |  |
| Licenced vaccine available | 1617 (100.0) | No (≤11 y) | 660 (70.4) | 372 (54.8) | <0.001 |
|  |  | Yes (≥ 12 y) | 278 (29.6) | 307 (45.2) |  |
| Sex at Birth | 1613 (99.8) | Male | 496 (52.9) | 367 (54.1) | 0.745 |
|  |  | Female | 438 (46.7) | 312 (45.9) |  |
|  |  | (Missing) | 4 (0.4) | 0 (0.0) |  |
| Ethnicity | 1385 (85.7) | White | 468 (49.9) | 334 (49.2) | 0.114 |
|  |  | Black | 49 (5.2) | 56 (8.2) |  |
|  |  | South Asian | 136 (14.5) | 97 (14.3) |  |
|  |  | Other ethnic minority | 147 (15.7) | 98 (14.4) |  |
|  |  | (Missing) | 138 (14.7) | 94 (13.8) |  |
| IMD quintile | 1491 (92.2) | 1 (most deprived) | 311 (33.2) | 231 (34.0) | 0.912 |
|  |  | 2 | 182 (19.4) | 128 (18.9) |  |
|  |  | 3 | 140 (14.9) | 91 (13.4) |  |
|  |  | 4 | 110 (11.7) | 85 (12.5) |  |
|  |  | 5 (least deprived) | 124 (13.2) | 89 (13.1) |  |
|  |  | (Missing) | 71 (7.6) | 55 (8.1) |  |
| Potential hospital acquired SARS-CoV-2 | 1617 (100.0) | No | 919 (98.0) | 631 (92.9) | <0.001 |
|  |  | Yes | 19 (2.0) | 48 (7.1) |  |
| Total PEWS | 1518 (93.9) | Median (IQR) | 2.0 (1.0 to 4.0) | 2.0 (1.0 to 5.0) | 0.014 |
| PEWS over 2 | 1518 (93.9) | No | 471 (50.2) | 351 (51.7) | 0.315 |
|  |  | Yes | 380 (40.5) | 316 (46.5) |  |
|  |  | (Missing) | 87 (9.3) | 12 (1.8) |  |
| Length of stay | 1368 (84.6) | Median (IQR) | 2.0 (1.0 to 3.0) | 3.0 (1.0 to 7.0) | <0.001 |

**Supplementary Table J.** Demographics and key clinical characteristics of CYP stratified by comorbidity (patients with asymptomatic or incidental SARS-CoV-2 infections excluded). Column percentages by sub-group. IMD = Indices of multiple deprivation. PEWS= Paediatric Early Warning Score at presentation. IQR = Interquartile range

|  | Total N |  | No/Unknown Comorbidity | Comorbidity  present | p |
| --- | --- | --- | --- | --- | --- |
| Total N (%) |  |  | 863 (56.6) | 663 (43.4) |  |
| Antibiotic medication | 1394 (91.3) | No | 297 (34.4) | 215 (32.4) | 0.058 |
|  |  | Yes | 464 (53.8) | 418 (63.0) |  |
|  |  | (Missing) | 102 (11.8) | 30 (4.5) |  |
| Antiviral | 1385 (90.8) | No | 719 (83.3) | 579 (87.3) | 0.008 |
|  |  | Yes | 35 (4.1) | 52 (7.8) |  |
|  |  | (Missing) | 109 (12.6) | 32 (4.8) |  |
| Maximal steroid therapy | 1350 (88.5) | None | 693 (80.3) | 468 (70.6) | <0.001 |
|  |  | Oral | 49 (5.7) | 102 (15.4) |  |
|  |  | IV | 8 (0.9) | 30 (4.5) |  |
|  |  | (Missing) | 113 (13.1) | 63 (9.5) |  |
| Maximum respiratory support | 1459 (95.6) | No respiratory support | 701 (81.2) | 418 (63.0) | <0.001 |
|  |  | Supplemental oxygen | 53 (6.1) | 106 (16.0) |  |
|  |  | High flow support | 17 (2.0) | 45 (6.8) |  |
|  |  | Non-invasive | 14 (1.6) | 35 (5.3) |  |
|  |  | Invasive | 16 (1.9) | 54 (8.1) |  |
|  |  | (Missing) | 62 (7.2) | 5 (0.8) |  |
| ICU/HDU admission | 1459 (95.6) | No | 744 (86.2) | 528 (79.6) | <0.001 |
|  |  | Yes | 59 (6.8) | 128 (19.3) |  |
|  |  | (Missing) | 60 (7.0) | 7 (1.1) |  |
| Inotrope | 1360 (89.1) | No | 729 (84.5) | 602 (90.8) | 0.002 |
|  |  | Yes | 7 (0.8) | 22 (3.3) |  |
|  |  | (Missing) | 127 (14.7) | 39 (5.9) |  |
| Total PEWS | 1427 (93.5) | Median (IQR) | 2.0 (1.0 to 4.0) | 2.0 (1.0 to 5.0) | 0.001 |
| PEWS over 2 | 1427 (93.5) | No | 447 (51.8) | 346 (52.2) | 0.102 |
|  |  | Yes | 329 (38.1) | 305 (46.0) |  |
|  |  | (Missing) | 87 (10.1) | 12 (1.8) |  |
| Length of stay | 1301 (85.3) | Median (IQR) | 2.0 (1.0 to 3.0) | 3.0 (1.0 to 6.0) | <0.001 |

**Supplementary Table K.** Treatments received stratified by comorbidity (excluding patients with asymptomatic or incidental SARS-CoV-2 infections and those with Multisystem Inflammatory Syndrome in Children (MIS-C)). ICU = intensive care unit. HDU = high dependency unit. PEWS= Paediatric Early Warning Score at presentation.

|  |  | Asympto / Incidental | Symptomatic | p |
| --- | --- | --- | --- | --- |
| Total N (%) |  | 427 (21.7) | 1540 (78.3) |  |
| Age at assessment (Years) | Median (IQR) | 11.2 (1.5 to 15.9) | 5.3 (0.4 to 14.2) | <0.001 |
| Age | <1 mth | 30 (7.0) | 111 (7.2) | <0.001 |
|  | >1mth <1 y | 54 (12.6) | 399 (25.9) |  |
|  | 1-4 y | 64 (15.0) | 246 (16.0) |  |
|  | 5-9 y | 49 (11.5) | 182 (11.8) |  |
|  | 10-14 y | 85 (19.9) | 263 (17.1) |  |
|  | 15-19 y | 145 (34.0) | 339 (22.0) |  |
| Sex at Birth | Male | 214 (50.1) | 826 (53.6) | 0.224 |
|  | Female | 212 (49.6) | 712 (46.2) |  |
|  | (Missing) | 1 (0.2) | 2 (0.1) |  |
| Ethnicity | White | 240 (56.2) | 778 (50.5) | 0.172 |
|  | Black | 27 (6.3) | 100 (6.5) |  |
|  | South Asian | 56 (13.1) | 218 (14.2) |  |
|  | Other ethnic minority | 51 (11.9) | 237 (15.4) |  |
|  | (Missing) | 53 (12.4) | 207 (13.4) |  |
| IMD quintile | 1 (most deprived) | 120 (28.1) | 515 (33.4) | 0.947 |
|  | 2 | 75 (17.6) | 299 (19.4) |  |
|  | 3 | 55 (12.9) | 219 (14.2) |  |
|  | 4 | 47 (11.0) | 188 (12.2) |  |
|  | 5 (least deprived) | 44 (10.3) | 203 (13.2) |  |
|  | (Missing) | 86 (20.1) | 116 (7.5) |  |
| Potential hospital acquired SARS-CoV-2 | No | 374 (87.6) | 1475 (95.8) | <0.001 |
|  | Yes | 53 (12.4) | 65 (4.2) |  |
| Total PEWS | Median (IQR) | 1.0 (0.0 to 2.0) | 2.0 (1.0 to 4.0) | <0.001 |
| Any comorbidity | No/Unknown | 208 (48.7) | 867 (56.3) | 0.006 |
|  | Yes | 219 (51.3) | 673 (43.7) |  |
| Alternative reason for admission | No / Unknown | 295 (69.1) | 1458 (94.7) | <0.001 |
|  | Yes | 132 (30.9) | 82 (5.3) |  |

**Supplementary Table L.** Demographics and key clinical characteristics of children with asymptomatic or incidental SARS-CoV-2 infections compared to those who were symptomatic. Column percentages by subgroup. IMD = Indices of multiple deprivation. PEWS= Paediatric Early Warning Score at presentation. IQR = Interquartile range

## Supplementary Figures


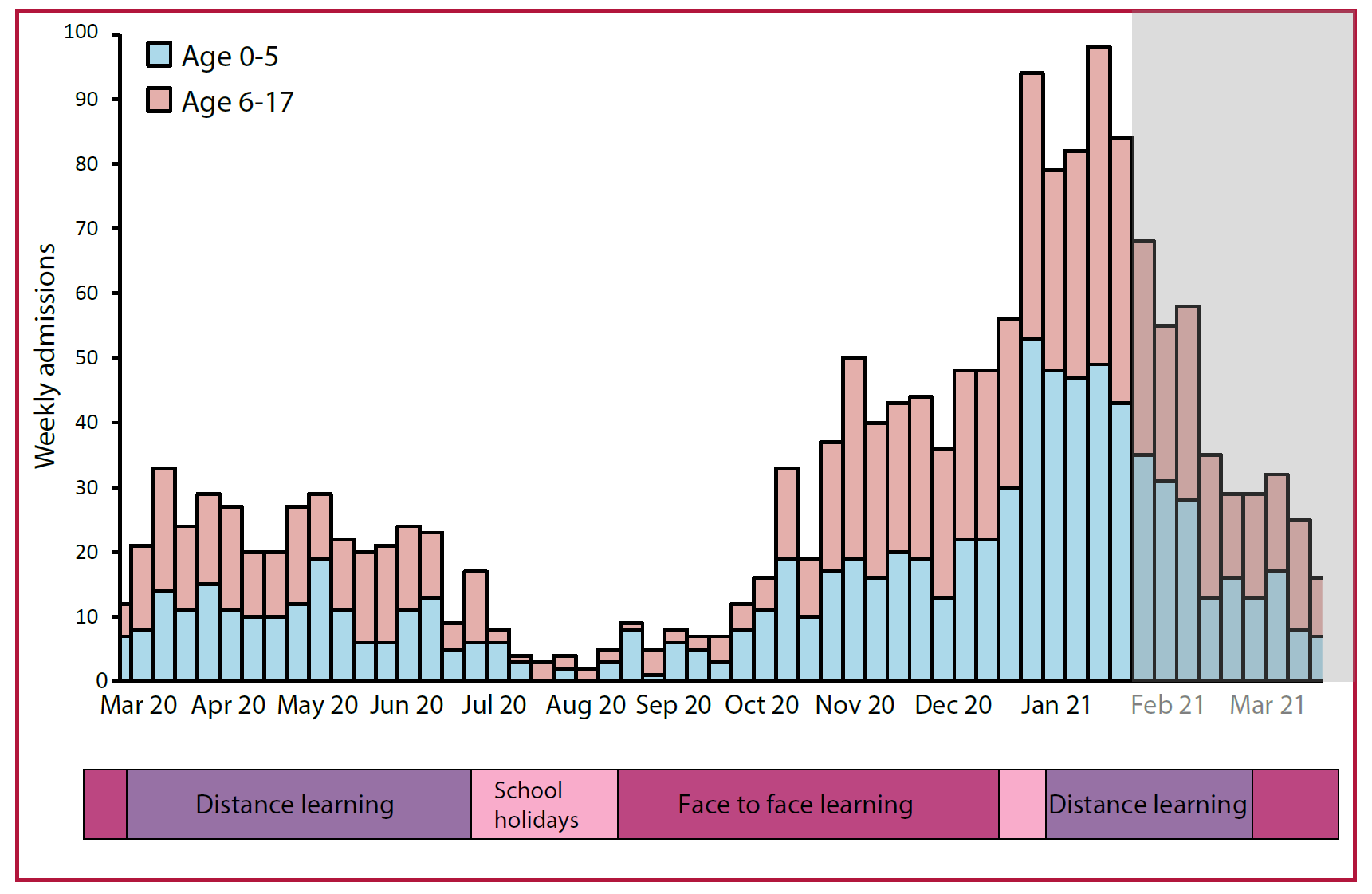


***Supplementary Figure A.*** *Timeline of major lockdown events in England against SARS-CoV-2 admissions for patients under 18 years across England (admissions data from NHS England).^4^*

*Magenta = face to face learning, purple = distance learning, pink = school holidays.*

*This ISARIC4C analysis spans the period indicated by a white background. (NB. ISARIC-4C also includes data from Scotland and Wales)*

*
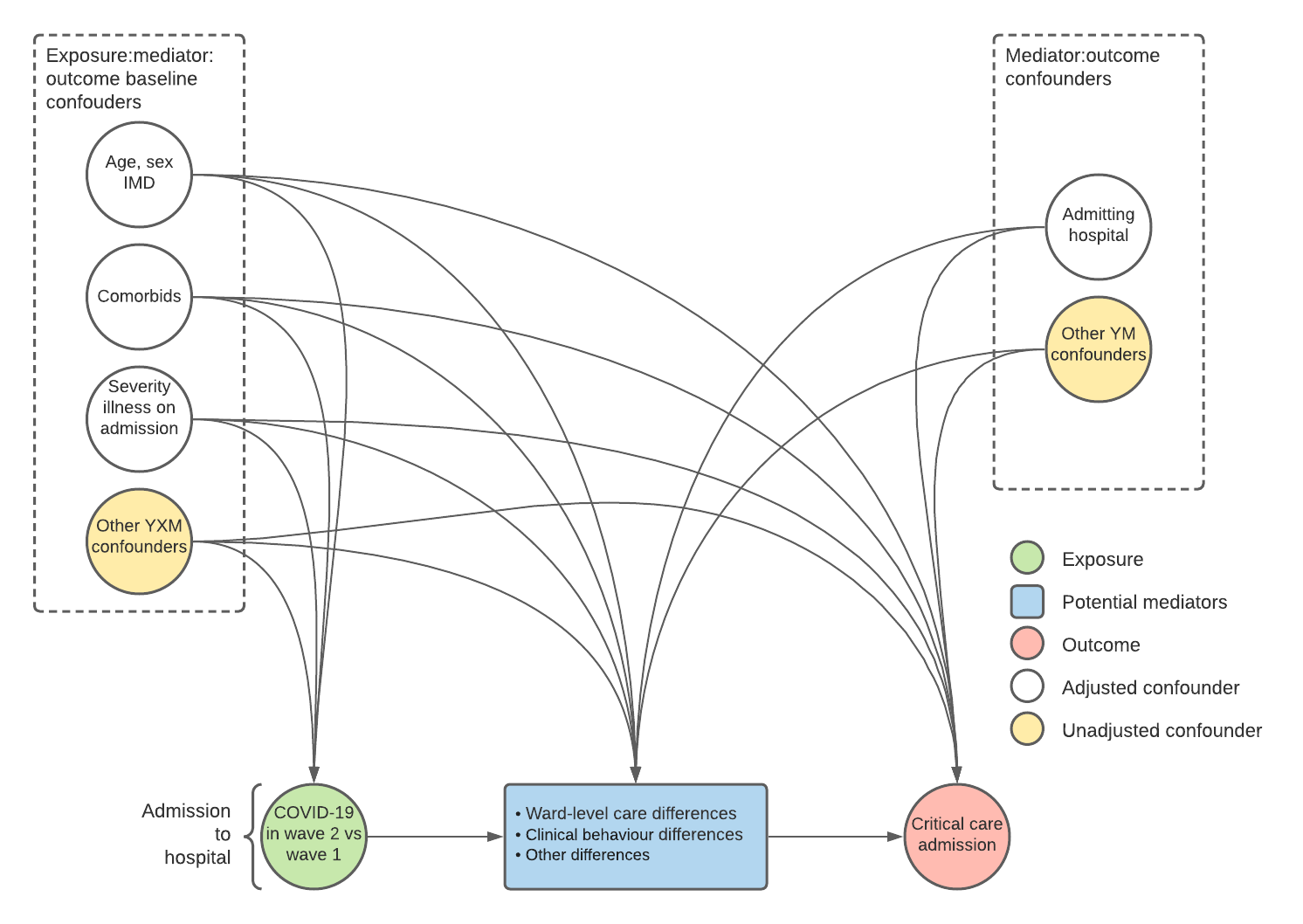
*

**Supplementary Figure B.** Directed acyclic graph of factors associated with critical care admission for construction of multivariable analysis


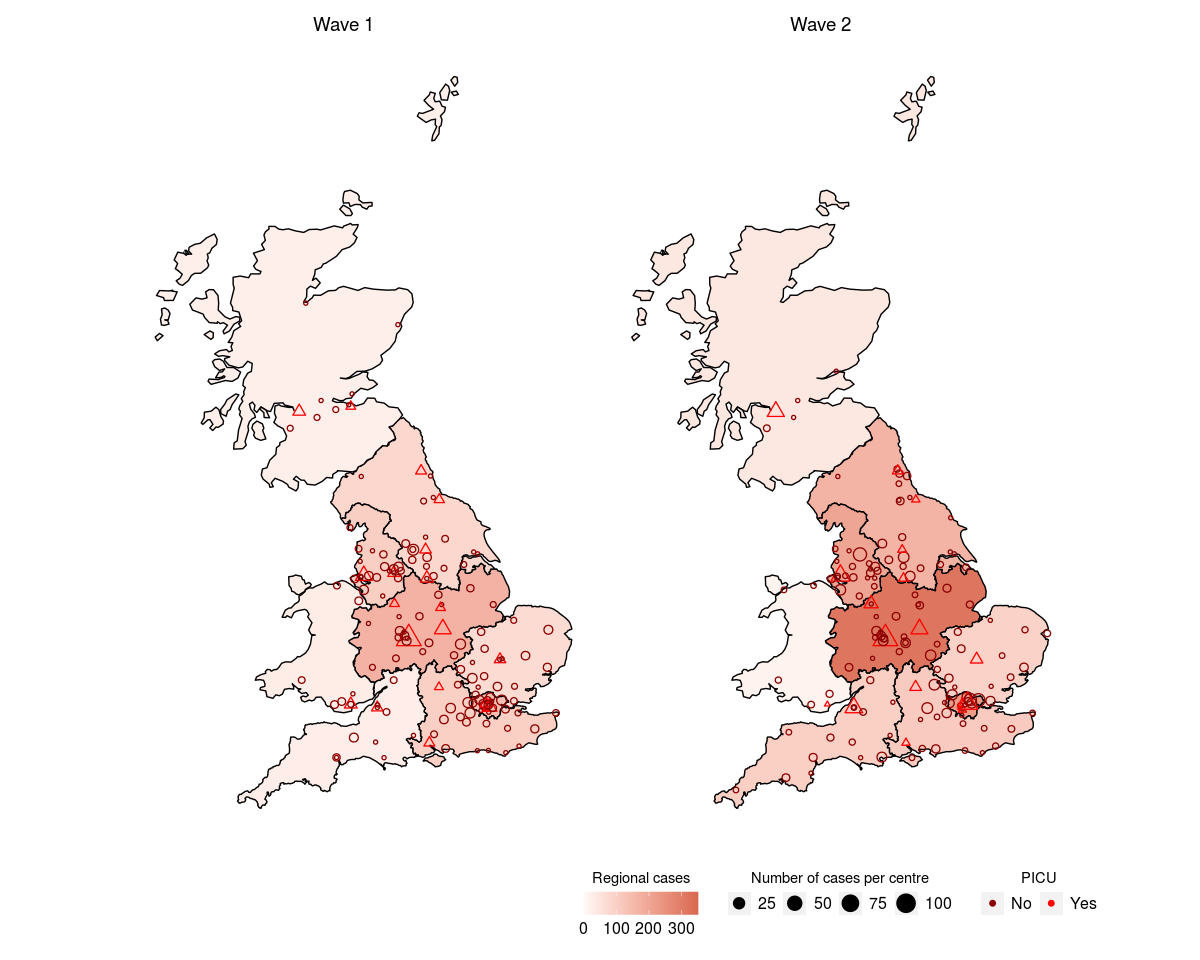


**Supplementary Figure C.** Map of sites of patient enrolment and cases by site. Sites with access to an onsite PICU are represented with triangles and those without as circles.


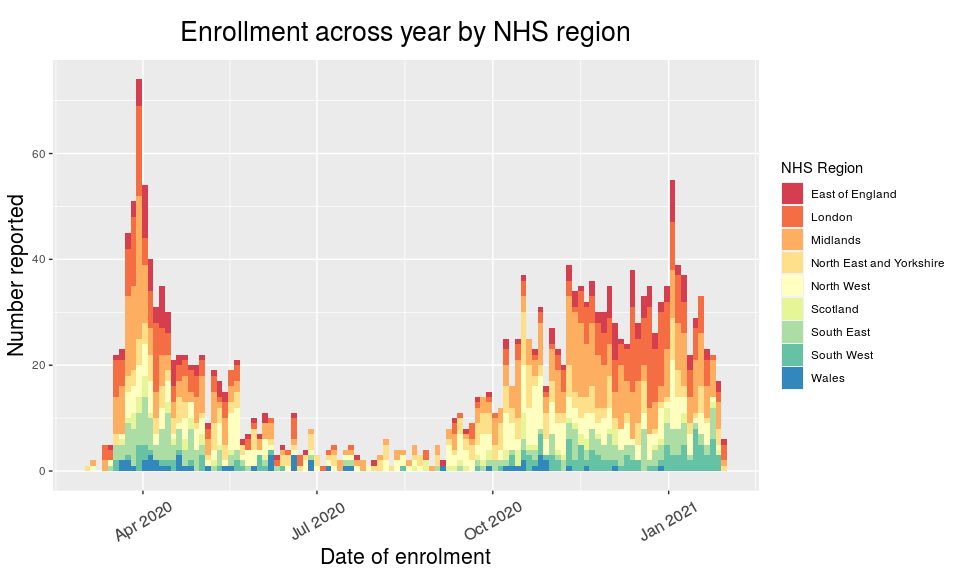


**Supplementary Figure D.** Reporting of patients <19 years to ISARIC4C by NHS region. Regional peaks can be seen in November in the Midland (orange) and London in December (dark orange). These peaks closely mirrored those reported by Public Health England at the same time points.^3^


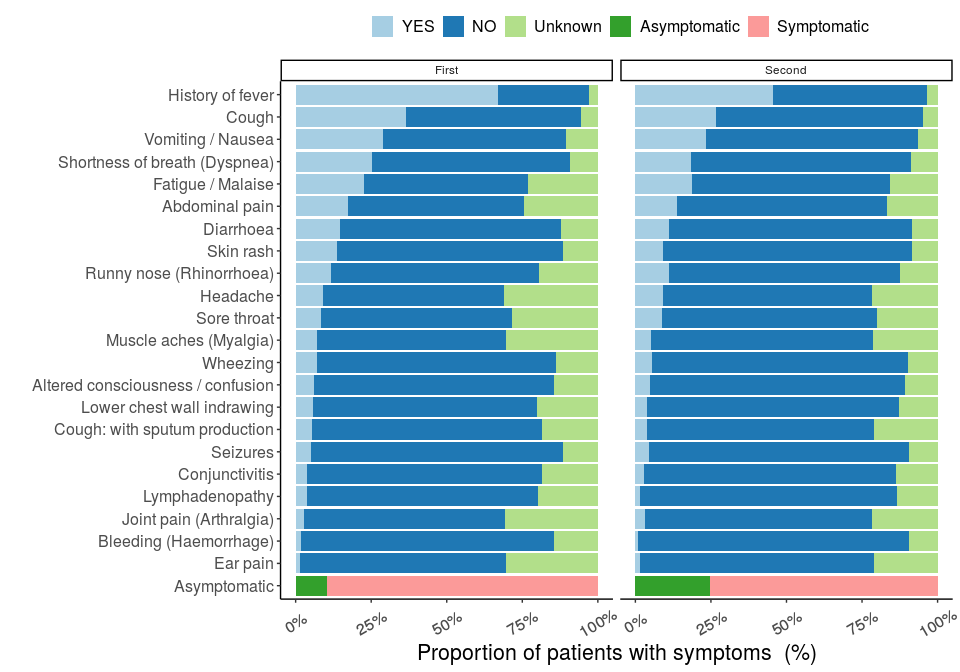


**Supplementary Figure E**. Comparison of symptoms at presentation (whole cohort analysis).


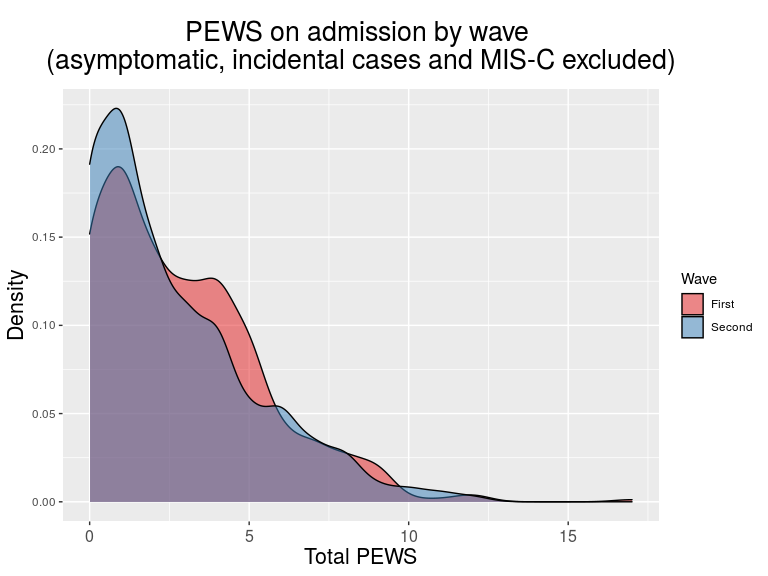


**Supplementary Figure F**. Paediatric Early Warning Score (PEWS) at presentation compared across the two waves (asymptomatic or incidental SARS-CoV-2 infections and patients with MIS-C excluded).


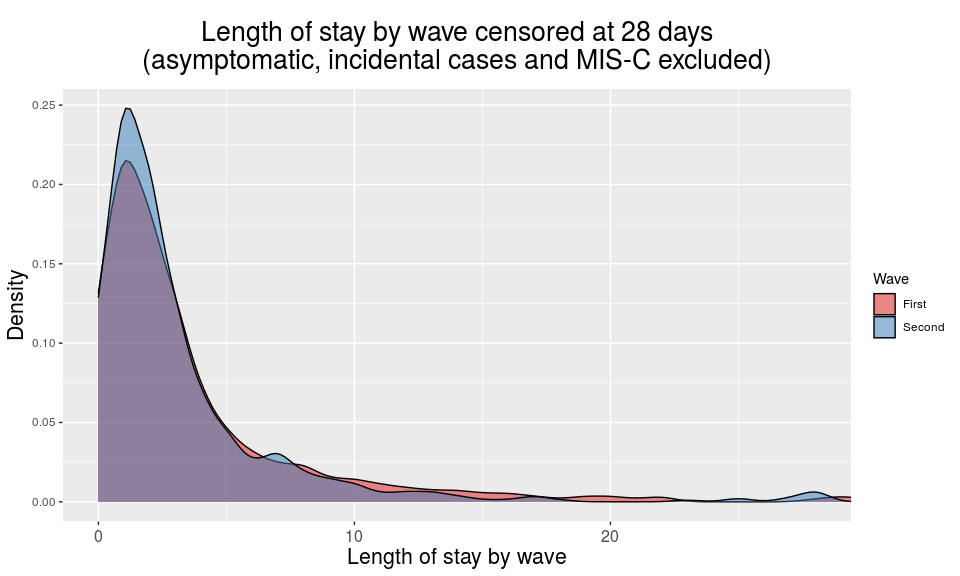


**Supplementary Figure G**. Length of stay compared across the two waves (asymptomatic / incidental SARS-CoV-2 infections and patients with MIS-C excluded)

*
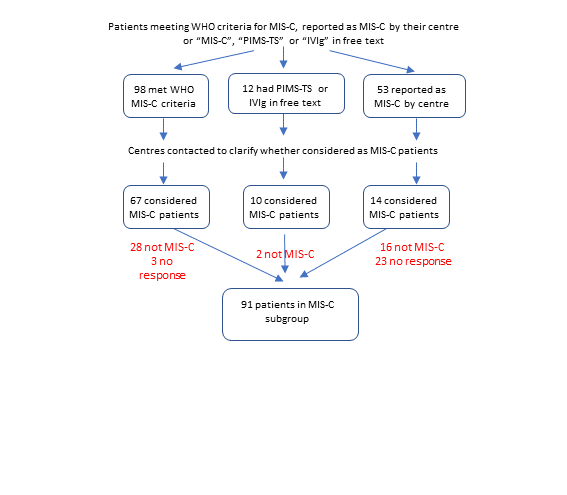
*

**Supplementary Figure H**. Flowchart for indentification of patients with multisystem inflammatory syndrome (MIS-C). WHO = World Health Organisation, PIMS-TS = Paediatric Multisystem Inflammatory Syndrome – Temporally Associated with SARS-CoV-2, IVIg = Intravenous immunoglobulin.

## References

1.Health Improvement Scotland. Paediatric Early Warning Score (PEWS) Charts. <https://ihub.scot/improvement-programmes/scottish-patient-safety-programme-spsp/spsp-programmes-of-work/maternity-and-children-quality-improvement-collaborative-mcqic/paediatric-care/pews/> (accessed 25/08/2021).

2.Swann OV, Holden KA, Turtle L, et al. Clinical characteristics of children and young people admitted to hospital with covid-19 in United Kingdom: prospective multicentre observational cohort study. *BMJ* 2020; **370**: m3249.

3.Public Health England. COVID-19 vaccine surveillance report: Week 20. May 2021. https://www.gov.uk/government/publications/covid-19-vaccine-surveillance-report (accessed 25/08/2021).

4.NHS England. COVID-19 Hospital Activity. https://www.england.nhs.uk/statistics/statistical-work-areas/covid-19-hospital-activity/ (accessed 25/08/2021).
